# TCF21 and AP-1 interact through epigenetic modifications to regulate coronary artery disease gene expression

**DOI:** 10.1186/s13073-019-0635-9

**Published:** 2019-05-02

**Authors:** Quanyi Zhao, Robert Wirka, Trieu Nguyen, Manabu Nagao, Paul Cheng, Clint L. Miller, Juyong Brian Kim, Milos Pjanic, Thomas Quertermous

**Affiliations:** 1 Division of Cardiovascular Medicine and Cardiovascular Institute, School of Medicine, Stanford University, 300 Pasteur Dr., Falk CVRC, Stanford, CA 94305 USA; 2 Center for Public Health Genomics, Department of Public Health Sciences, University of Virginia, Charlottesville, VA 22908 USA; 3 Center for Public Health Genomics, Biochemistry and Molecular Genetics, University of Virginia, Charlottesville, VA USA; 4 Center for Public Health Genomics, Biomedical Engineering, University of Virginia, Charlottesville, VA USA

**Keywords:** Transcription, Epigenomics, TCF21, AP-1, Histone acetyltransferase, Deacetylase

## Abstract

**Background:**

Genome-wide association studies have identified over 160 loci that are associated with coronary artery disease. As with other complex human diseases, risk in coronary disease loci is determined primarily by altered expression of the causal gene, due to variation in binding of transcription factors and chromatin-modifying proteins that directly regulate the transcriptional apparatus. We have previously identified a coronary disease network downstream of the disease-associated transcription factor TCF21*,* and in work reported here extends these studies to investigate the mechanisms by which it interacts with the AP-1 transcription complex to regulate local epigenetic effects in these downstream coronary disease loci.

**Methods:**

Genomic studies, including chromatin immunoprecipitation sequencing, RNA sequencing, and protein-protein interaction studies, were performed in human coronary artery smooth muscle cells.

**Results:**

We show here that TCF21 and JUN regulate expression of two presumptive causal coronary disease genes, *SMAD3* and *CDKN2B-AS1*, in part by interactions with histone deacetylases and acetyltransferases. Genome-wide TCF21 and JUN binding is jointly localized and particularly enriched in coronary disease loci where they broadly modulate H3K27Ac and chromatin state changes linked to disease-related processes in vascular cells. Heterozygosity at coronary disease causal variation, or genome editing of these variants, is associated with decreased binding of both JUN and TCF21 and loss of expression in *cis*, supporting a transcriptional mechanism for disease risk.

**Conclusions:**

These data show that the known chromatin remodeling and pioneer functions of AP-1 are a pervasive aspect of epigenetic control of transcription, and thus, the risk in coronary disease-associated loci, and that interaction of AP-1 with TCF21 to control epigenetic features, contributes to the genetic risk in loci where they co-localize.

**Electronic supplementary material:**

The online version of this article (10.1186/s13073-019-0635-9) contains supplementary material, which is available to authorized users.

## Background

Collaborative efforts from multiple large genome-wide association studies (GWAS) in the past decade have identified 161 independent genomic loci significantly associated with CAD in humans [1–4]. While a small subset of coronary artery disease (CAD) variants alter specific protein composition, the majority of the identified lead SNPs (single nucleotide polymorphisms) are located in non-coding enhancer regions, suggesting that variation in cellular transcriptional response rather than protein function underlies the majority of heritable disease risk [5]. Although identified genetic loci offer great promise in advancing our understanding of CAD pathophysiology, these transcriptional regulatory regions are poorly annotated, and thus, the mechanisms by which the causal variants affect causal gene expression are poorly understood. Identifying the transcriptional mechanisms shared among multiple CAD risk loci will not only provide new insights into the pathophysiology of CAD, but will also allow us to identify core mechanisms for intervention to modify disease risk.

TCF21 is a basic helix-loop transcription factor critical in cardiovascular development and disease [2–4, 6–14]. In the context of atherosclerosis, an important feature of TCF21 biology is the fact that it regulates fundamental cellular differentiation events in the developing epicardium, serving as a determining factor for the divergence between coronary vascular smooth muscle cell (SMC) and cardiac fibroblast lineages [15, 16]. Embryonic loss of *Tcf21* has been linked to premature differentiation of SMC from the pericardium, resulting in decreased migration into the myocardium [16]. *TCF21* has been extensively associated with CAD risk, having been associated with disease in multiple racial ethnic groups [14, 17, 18]. Vascular smooth muscle cell expression quantitative trait locus (eQTL) studies [19], as well mechanistic follow-up studies, have identified *TCF21* as the causal gene in the CAD-associated locus at 6q23.2 [20] and suggested that expression of this gene is protective toward CAD risk [21]. Further, *TCF21* has been shown to be expressed in cells that migrate into the developing plaque and contribute to the protective fibrous cap [22]. TCF21 downstream target regions are enriched for known CAD risk loci, suggesting that TCF21 plays a central role in regulating risk in other loci to effect the biology of atherosclerotic plaques [23].

The activated protein-1 (AP-1) family of proteins includes a number of bZip transcription factors that bind primarily as heterodimers to a well-characterized canonical DNA sequence. Expression of these early response genes (e.g., *FOS*, *FOSB*, *JUN*, *JUND*) is initiated in all cell types when growth factors activate their cognate receptor tyrosine kinases, inducing the Ras/MAPK intracellular kinase cascade, promoting a variety of cell state changes [24–26]. These AP-1 site-binding proteins have been shown to play a central role in specific lineage-restricted enhancer selection and function [27, 28]. FOS/JUN factors select enhancers with cell type-specific transcription factors (TFs) by collaboratively binding to histone bound enhancers and recruiting the BAF chromatin remodeling complex to establish accessible chromatin [29]. At a more general level, AP-1 factors have been shown to disrupt the nucleosomal structure and regulate chromatin accessibility in various cell types [29–31]. In the context of complex diseases, variation in AP-1 binding sites has been shown to mediate genetic risk [20, 32–35]. An AP-1 site in an intron of the *SMAD3* gene has been implicated in the association of this gene with CAD, as well as an autoimmune disease [20, 33–35]. Important for this discussion, AP-1 binding sites in two different CAD-associated alleles that regulate expression of *TCF21* have been shown to alter risk in Caucasian and East Asian populations [14, 17, 20].

In studies reported here, we have investigated the interaction of transcription factors TCF21 and AP-1 at loci identified by GWAS to regulate risk for the CAD phenotype, focusing on 15q22.33 and 9p21.3. Studies employing eQTL mapping, allele-specific expression, co-localization of eQTL and GWAS, in vitro experiments, cell culture, and animal models have identified SMAD3 [19, 33, 36, 37] and *CDKN2B-AS1* (*CDKN2BAS*) [38–42] as putative causal genes in these loci. We show that TCF21 and AP-1 regulate expression of these genes by regulating chromatin accessibility through interactions with histone acetyltransferases and histone deacetylases and also through protein-protein interactions. We subsequently generalize our finding on these two loci genome-wide and demonstrate that interaction between AP1 and TCF21 controls epigenetic features in regions where they co-localize. These regions are enriched for CAD genetic risk loci, suggesting a critical role for this interaction in modifying risk for CAD.

## Methods

### Primary cell culture and reagents

Primary human coronary artery smooth muscle cells (HCASMCs) derived from normal human donor hearts were purchased from three different manufacturers Lonza, PromoCell, and Cell Applications (all tested negative for mycoplasma contamination) at passage 2 and were maintained in growth-supplemented smooth muscle basal media (Lonza # CC-3182) according to the manufacturer’s instructions. All experiments were performed on HCASMCs between passages 4 and 7. Antibodies used for ChIPseq and ChIP-quantitative PCR (qPCR) were all pre-validated according to ChIPseq guidelines and ENCODE best practices. Purified mouse monoclonal antibodies against human JUN (sc-74543) and p300 (sc-48343) were purchased from Santa Cruz. Purified rabbit polyclonal antibody against H3K27ac (ab4729), HDAC1 (ab7028), and HDAC2 (ab7029) were purchased from Abcam. Purified rabbit polyclonal antibody against human TCF21 (HPA013189) was purchased from Sigma.

### Knockdown and overexpression

*JUN* (s7659) and p300 (s534247) silencer select siRNAs were purchased from Thermo Fisher Scientific. *TCF21* (SR321985) Trilencer-27 siRNAs were purchased from OriGene. siRNA transfection was performed using Lipofectamine RNAiMAX (Life Technologies). For each well treated with the siRNAs or scrambled control (Life Technologies, #4390843), the final concentration was 20 nM. HCASMCs were seeded in 6-well plates and grown to 50% confluence before siRNA transfection. HCASMCs were transfected with the siRNA or scrambled control for 6–8 h and subsequently collected and processed for RNA isolation after 48 h of transfection using the RNeasy mini kit (Qiagen 74106).

For lentivirus transduction study, control (titer 5.68 × 10^7^), *TCF21* cDNA (titer 3.84 × 10^6^), and shRNA (titer 2.59 × 10^7^) virus were used as described before [22]. *JUN* shRNA (titer 8.4 × 10^8^) virus was purchased from OriGene (TL320397). HCASMCs were transduced with the virus at a density of 5 × 10^5^ cells per T75-flask. Cells were changed to medium with supplements 12 h after transduction and cultured for an additional 48 h. The transduction efficiency was assessed by quantifying the percentage of GFP-positive cells. Transduction efficiencies were more than 90%.

### RNA isolation and qRT-PCR

RNA for all samples was extracted using the RNeasy mini kit (Qiagen 74106). HCASMC RNA (500 ng) was reverse transcribed using the High-Capacity RNA-to-cDNA Synthesis kit (Applied Biosystems 4387406). Quantitative PCR of the cDNA samples was performed on a ViiA7 Real-Time PCR system, and gene expression levels were measured using SYBR green assays (Applied Biosystems 4368706) using custom-designed primers and normalized to *ACTB* levels. Comparison between two groups was performed using the Student’s *t* test, and comparisons between the three groups were performed using *ANOVA*.

qPCR primers were designed as follows: *SMAD3*, Fwd: CCATCTCCTACTACGAGCTGAA, Rev: CACTGCTGCATTCCTGTTGAC, Amplicon: 149 bp; *CDKN2BAS*, Fwd: CTATCCGCCAATCAGGAGGC, Rev: GCGTGCAGCGGTTTAGTTTA, Amplicon: 103 bp; *CDKN2B*, Fwd: GGGACTAGTGGAGAAGGTGC, Rev: CATCATCATGACCTGGATCGC, Amplicon: 97 bp; *JUN*, Fwd: TCCAAGTGCCGAAAAAGGAAG, Rev: CAGCACAATGAAGATCAAGA, Amplicon: 78 bp; *TCF21*, Fwd: TCCTGGCTAACGACAAATACGA, Rev: TTTCCCGGCCACCATAAAGG, Amplicon: 77 bp; *EP300*, Fwd: GCTTCAGACAAGTCTTGGCAT, Rev: ACTACCAGATCGCAGCAATTC, Amplicon: 79 bp.

### ChIP assay

Briefly, approximately 4e6 HCASMC cells were fixed with 1% formaldehyde and quenched by glycine. The cells were washed three times with PBS and then harvested in ChIP lysis buffer (50 mM Tris-HCl, pH 8, 5 mM EDTA, 0.5% SDS). Crosslinked chromatin was sheared for 3 × 1 min by sonication (Branson SFX250 Sonifier) before extensive centrifugation. Four volumes of ChIP dilution buffer (20 mM Tris-HCl, pH 8.0, 150 mM NaCl, 2 mM EDTA, 1% Triton X-100) was added to the supernatant. The resulting lysate was then incubated with Dynabeads™ Protein G (Thermo Scientific, 10009D) and antibodies at 4 °C overnight. Beads were washed once with buffer 1 (20 mM Tris pH 8, 2 mM EDTA, 150 mM NaCl, 1% Triton X100, 0.1% SDS), once with buffer 2 (10 mM Tris pH 8, 1 mM EDTA, 500 mM NaCl, 1% Triton X100, 0.1% SDS), once with buffer 3 (10 mM Tris pH 8, 1 mM EDTA, 250 mM LiCl, 1% NP40, 1% sodium deoxycholate monohydrate), and twice with TE buffer. DNA was eluted by ChIP elution buffer (0.1 M NaHCO_3_, 1% SDS, 20 μg/ml proteinase K). The elution was incubated at 65 °C overnight, and DNA was extracted with a DNA purification kit (Zymo D4013). The purified DNA was assayed by quantitative PCR with ABI ViiA 7 and Power SYBR Green Master Mix (ABI 4368706). For serial ChIP, the DNA-protein complex of the first IP was eluted with elution buffer containing no proteinase K after washing. The elution was diluted 1:9 by dilution buffer followed by the second IP and washing steps. Heterozygous genotypes at the candidate loci were determined using TaqMan SNP genotyping qPCR assays (Thermo Fisher Scientific C__33991343_10). Assays were repeated at least three times. Data shown were average values ± SD of representative experiments.

qPCR primers were designed as follows: *SMAD3-1*, Fwd: GGTTGACCCGTTGCATGTTA, Rev: GGAGAGGTGAAGAGGGCAAA, Amplicon: 129 bp; *SMAD3-2*, Fwd: AGAGGGCAGAGAGAGGATAC, Rev: AGTCTTATCTGCCGGCAAAC, Amplicon: 142 bp; *CDKN2BAS*, Fwd: GCATTGAGAAGTCCAGCCAG, Rev: GCAGCAACTTCGAAGCTTGA, Amplicon: 136 bp.

### Immunoprecipitation and western blotting

Nuclear complex Co-IP kit (Active Motif 54001) was used for co-immunoprecipitation following the manufacturer instructions. ChIP samples were eluted with 1× Laemmli buffer (Bio-Rad 161-0747) containing Halt protease inhibitor cocktail (Thermo Fisher Scientific 78429) instead of elution buffer for western blotting. The elution was incubated at 65 °C for 6 h and then boiled at 95 °C for 2 min. Whole cell lysate samples were harvested at 4 °C using 1× Laemmli buffer and boiled at 95 °C for 10 min.

The 5–40-μl sample was loaded onto a 4–15% gradient SDS-PAGE gel (Bio-Rad 4561084DC). Samples were transferred to polyvinylidene difluoride membrane (Thermo Fisher Scientific LC2002) for 1 h at 300 mA at 4 °C and blocked with 5% milk in Tris-buffered saline and 0.1% Tween 20 (TBST, Bio-Rad 1706435) at room temperature. The membranes were hybridized with the following primary antibodies: mouse Myc-Tag antibody (CST 2276S), rabbit SMAD3 antibody (CST 9523S), rabbit c-JUN antibody (CST 9165S), p300, HDAC1, and HDAC2 antibodies as described above. Rabbit GAPDH antibody (Sigma G9545-200UL) was used as the loading control. Anti-mouse HRP (Jackson ImmunoResearch 115-035-008, 115-035-003, and 115-035-174) or anti-rabbit HRP (Jackson ImmunoResearch 211-032-171, 111-035-008, and 111-035-003) secondary antibodies were used at a concentration of 1:10000 and diluted in 5% milk containing 0.1% Tween 20. Bands were detected using SignalBoost™Immunoreaction Enhancer Kit (Millipore 407207) per manufacturer’s instructions on the LI-COR Odyssey imaging system.

### ChIPseq analysis

ChIP was performed with approximately 4xE6 HCAMSC cells using H3K27ac antibody (Abcam ab4729) under *JUN* siRNA or *TCF21* shRNA knockdown described in the “Knockdown and overexpression” section. Two biological replicate DNA were combined for library preparation. Libraries were prepared with KAPA Hyper Prep kit (KK8502). ChIPseq libraries were sequenced on HiSeq X10 for 150 bp paired-end sequencing. Quality control of ChIPseq data was performed using *Fastqc*, and then low-quality bases and adaptor contamination were trimmed by *cutadapt*. Filtered reads were mapped to hg19 using *BWA mem* algorithm. Duplicate reads were marked by *Picard Markduplicate* module and removed with unmapped reads by *samtools view -f 2 -F 1804*. *macs2 callpeak* was used for peaks calling with *--broad* parameters and input as control. *macs2 bdgdiff* was used for differential peaks calling with default parameters. The fastq files of TCF21 (SRR1573744&SRR1573745), JUN (SRR1573749), and HNF1A (SRR5339317) ChIPseq were extracted from GEO database by *fastq-dump*. Similar methods (except *--broad* parameter in *macs2*) were used in quality control, alignment, filtering, and peak calling. The average fragment size for the JUN ChIPseq was 186 bp, and for TCF21 was 196 bp.

### ATACseq analysis

Approximately 5xE4 fresh HCAMSC cells, each with *TCF21* shRNA knockdown or lentivirus overexpression, and *JUN* shRNA knockdown (described in the “Knockdown and overexpression” section) were collected by centrifugation at 500 g and washed twice with cold PBS. Nuclei-enriched fractions were extracted with cold resuspension buffer (0.1% NP-40, 0.1% Tween 20, and 0.01% Digitonin) and washed out with 1 ml of cold resuspension buffer containing 0.1% Tween 20 only. Nuclei pellets were collected by centrifugation and resuspended with transposition reaction buffer containing Tn5 transposases (Illumina Nextera). Transposition reactions were incubated at 37 °C for 30 min, followed by DNA purification using the DNA Clean-up and Concentration kit (Zymo D4013). Libraries were amplified using Nextera barcodes and high-fidelity polymerase (NEB M0541S) and purified using Agencourt Ampure XP beads (Beckman Coulter A63880) double-size selection (0.5X:0.9X). For qPCR experiments, the purified DNA was quantified with ABI ViiA 7 and Power SYBR Green Master Mix (ABI 4368706) and normalized by genomic DNA which extracted using Quick-DNA Microprep Kit (Zymo D3020). Assays were repeated at least three times. Data shown were average values ± SD of representative experiments.

Libraries were sequenced on HiSeq X10 for 150-bp paired-end sequencing. Raw fastq files were evaluated with *fastqc,* and then low-quality bases and adaptor contamination were trimmed by *cutadapt*. Reads were mapped to hg19 using *bowtie2*. Duplicate reads were marked by *Picard Markduplicate* module and removed with unmapped or mitochondrial reads by *samtools*. *bedtools* was used to generate BED file from filtered reads followed by Tn5 shifting with *awk*. *macs2 callpeak* with *--broad* and *SICER-rb.sh* with *“W200 G600 E1000*” parameters were used for peak calling. *macs2 bdgdiff* and *SICER-df-rb.sh* with *FDR* cutoff 0.05 were used for differential peak comparison in *JUN* or *TCF21* disrupted samples. Bigwig files were generated for University of California Santa Cruz (UCSC) Genome Browser visualization.

### Peak co-localization and enrichment analysis

The *intersectBed* was used to find at least 1 bp overlapped peaks between H3K27ac, ATAC, TCF21, and JUN. We combined the open chromatin regions from ENCODE data as intersection background. *P* values were calculated by *Fisher’s exact test*. For the common (conserved) open chromatin of ENCODE, we defined the peaks which have overlaps in more than 50% of the cell lines (70 out of 126 lines) that are conserved. These peaks in each line were merged to get the final common open chromatin regions. For enrichment level analysis, reads of control/treatment samples which mapped on peaks were counted by *intersectBed* and normalized by per million (RPM). JUN, TCF21 peaks, or open chromatin regions were clustered using the *hierarchical* algorithm, and reads centering on these peaks (± 2 kb) were plotted with *deeptools*.

### Cis-regulatory functional enrichment and network analysis

We utilized the *Genomic Regions Enrichment of Annotations Tool (GREAT 3.0)* to analyze the detected peaks, with the parameter “single nearest gene,” which is within 50 kb to nearest genes. Gene ontology from *GREAT* output was analyzed by *DAVID*. KEGG pathways, biological processes, and GAD disease enrichment analysis was carried out using default settings. The *HOMER findMotifsGenome.pl* script was employed to search for known TRANSFAC motifs and to generate de novo motifs. *PWMScore* was used for position weight matrix scan. We obtained full-length JUN (MA0488.1) and TCF21 (MA0832.1) motifs from JASPAR.

### Association of CAD loci and GWAS enrichment analysis

HCAMSC eQTL data came from a genome-wide association of gene expression with imputed common variation identified in 52 HCASMC lines studied with whole genome RNA sequencing and 30× whole-genome sequencing [19]. CARDIoGRAMplusC4D variant data was from 1000 Genome-based GWAS meta-analyses [43]. Coronary artery (CA) tissue-specific SNP gene association data was obtained from GTEx V6p. We intersected open chromatin region which regulated by TCF21 and AP-1 with these three datasets; the significance cutoff for GTEx is *nominal cis*-eQTL *P* < 0.05, *FDR* < 0.05 for HCAMSC eQTL, and *beta P* < 0.05 for CARDIoGRAMplusC4D. Overlap of TCF21-AP1 co-regulated open chromatin region and GWAS Catalog SNPs was performed with *bed2GwasCatalogBinomialMod1Ggplot* script from *gwasanalytics* package. The calculation criteria of this script were described previously [44].

### Statistical analysis

All experiments were performed by the investigators blinded to the treatments/conditions during the data collection and analysis, using at least two independent biological replicates and treatments/conditions in technical triplicate. *R* or *GraphPad Prism* was used for statistical analysis. For motifs and gene enrichment analyses, we used the *cumulative binomial distribution test*. For overlapping of genomic regions or gene sets, we used *Fisher’s exact test*. For comparisons between two groups of equal sample size (and assuming equal variance), an *unpaired two-tailed Student’s t test* was performed or in cases of unequal sample sizes or variance, *Welch’s unequal variances t test* was performed, as indicated. *P* values < 0.05 were considered statistically significant. For multiple comparison testing, *one-way analysis of variance (ANOVA)* accompanied by *Tukey’s* post hoc *test* were used as appropriate. All error bars represent standard error of the mean (SE). Number of stars for the *P* values in the graphs: ****P < 0.0001, ***P < 0.001, **P < 0.01, *P < 0.05.

### Generation and analysis of CRISPR lines

HEK293 cell line was used for genome editing. Cells were maintained in DMEM containing 10% FBS prior to and during transfection. Cells were seeded into 6-well plates at 2 × 10^5^ cells/well and were transfected with 2 μg sgRNA/Cas9-GFP using 7.5 μl Lipofectamine 3000 and 4 μl P3000 reagent per well 24 h after seeding. Two days after transfection cells were sorted using a Digital Vantage (BD Biosciences). GFP-positive cells were singly sorted into two 96-well plates and allowed to grow until clones reached about 70% confluence, about 2 weeks. All wells with colonies were marked and then split 1:2 into two fresh 96-well plates. Once a majority of the wells obtained 80% confluence, one plate of cells was frozen, and the other was used for genomic DNA extraction using Qiagen DNeasy Blood and Tissue Kit as per manufacturer’s instructions. The targeted region was amplified using 10 μl of gDNA, specific primers *SMAD3*_650F, and ChipSeqR and Taq DNA Polymerase (NEB) as per manufacturer’s instructions. Cycling conditions were as follows: 94 °C for 3 min, 35 cycles of 94 °C for 1 min, 63 °C for 30 s, 72 °C for 45 s, and one cycle of 72 °C for 5 min. All wells showing a positive band at about 500 bp were Sanger sequenced with *SMAD3*_650F primer, and the sequence was analyzed for indels. The methods of RNA extraction and expression analysis were described above.

### RNA seq analysis

HCASMC were transfected with *TCF21* or scrambled siRNA using the method described in the “Knockdown and overexpression” for three biological replicates. The cells were then collected 48 h after transfection and processed using RNeasy kit (Qiagen 74106) for RNA isolation. The cells were sent to Novogene for sample QC, library preparation, and sequencing. All samples passed QC, and 250–300 bp insert cDNA libraries were prepared for each sample. Subsequently, sequencing was performed on a Novaseq 6000 platform with paired-end 150 bp reads. The script for RNAseq analysis can be found in github (https://github.com/milospjanic/rnaSeqFPro). Briefly, QC of sequencing data was performed using *fastqc*. Then, *STAR* was used to map the reads to the reference genome (hg19). FeatureCounts from the *Subread* package was then used to count the number of reads mapped, and *deseq2* was used to generate the list of differentially regulated genes.

## Results

### JUN and TCF21 regulate CAD candidate causal gene expression in HCASMC

**Fig. 1:**
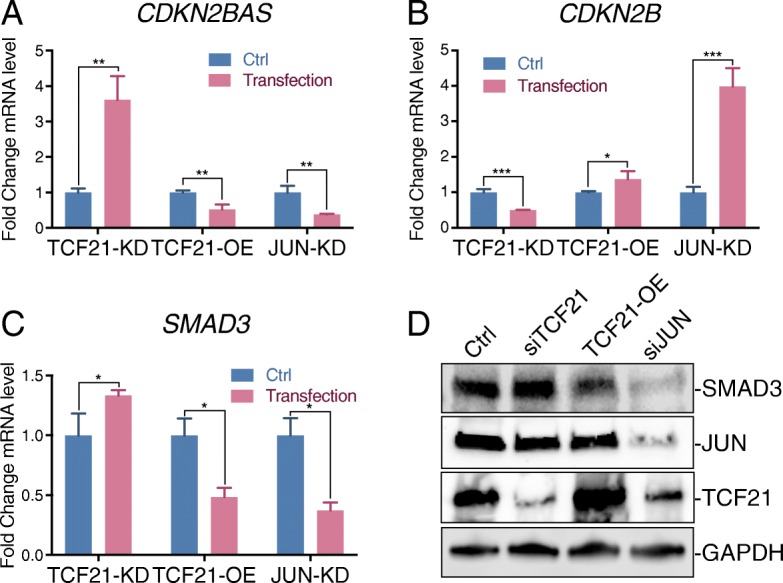
JUN and TCF21 regulate expression levels of CAD genes *SMAD3* and *CDKN2BAS/CDKN2B* in HCASMC. **a** HCASMC were transfected with *JUN* (JUN-KD), *TCF21* (TCF21-KD), scrambled (Ctrl) siRNA molecules, or transduced with empty pWPI vector (Ctrl) or a human *TCF21* cDNA clone (TCF21-OE) virus. The mRNA expression level of *CDKN2BAS* was evaluated by qPCR with *ACTB* normalization. **b**, **c** *CDKN2B* and *SMAD3* mRNA expression levels were quantified under identical conditions in HCASMC (mean ± SD, *n* = 3). **d** SMAD3, JUN, and TCF21 protein levels were evaluated by western blot, with GAPDH as loading control

AP-1 and TCF21 are regulatory transcription factors in human coronary artery smooth muscle cells (HCASMC) in a number of CAD loci, including *SMAD3* and *9p21* [20, 33]. To investigate the specific roles of AP-1 and TCF21 in regulating CAD gene transcription, we first focused our investigation on two candidate causal genes, *CDKN2BAS and SMAD3* [33, 38]. siRNA knockdown of *JUN* in HCASMC was associated with decreased expression levels of *CDKN2BAS and SMAD3* (Fig. 1a, c, Additional file 1: Figure S1A). On the other hand, knockdown of *TCF21* produced an opposite effect compared to *JUN* knockdown, producing increases in *SMAD3* and *CDKN2BAS* levels (Fig. 1a, c, Additional file 1: Figure S1A). *TCF21* overexpression produced results opposite to knockdown (Fig. 1a, c, Additional file 1: Figure S1B). Given the known anti-sense effect of *CDKN2BAS* [45], changes in *CDKN2BAS* expression were mirrored with opposite effects for the mRNA level of *CDKN2B* (Fig. 1a, b). To confirm these gene expression changes, we investigated the protein level of SMAD3 by western blotting. The SMAD3 protein level decreased with *JUN* knockdown or *TCF21* overexpression and increased with *TCF21* knockdown, consistent with mRNA levels (Fig. 1d, Additional file 1: Figure S1C). These data suggest that AP-1 serves as a transcriptional activator, and TCF21 acts as a suppressor of the presumptive CAD causal genes *CDKN2BAS and SMAD3*.

### JUN increases H3K27ac level and chromatin accessibility to promote TCF21 binding in SMAD3 and CDKN2BAS CAD loci

**Fig. 2:**
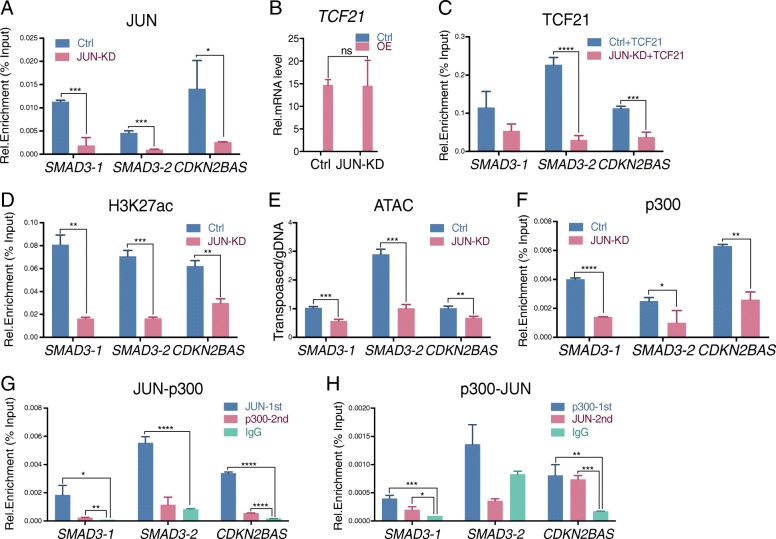
JUN recruits p300, promotes H3K27ac histone modification, and increases chromatin accessibility and TCF21 binding. **a**–**f** ChIP-qPCR at *SMAD3-1, SMAD3-2*, *and CDKN2BAS* locus regions with input normalization in HCASMC. **a** JUN binding was evaluated under conditions of *JUN* (JUN-KD) and scrambled (Ctrl) siRNA transfections. **b** Validation of overexpressed *TCF21* mRNA level under conditions of JUN-KD and Ctrl siRNA transfections by RT-qPCR. **c** pWI lentivirus expressed TCF21 binding was evaluated under conditions of JUN-KD or Ctrl siRNA transfection. **d** H3K27ac level was evaluated under conditions of JUN-KD or Ctrl siRNA transfection. **e** Chromatin accessibility assessed by ATAC-qPCR was evaluated under conditions of JUN-KD or Ctrl siRNA transfection. **f** p300 binding at *SMAD3-1/SMAD32 and CDKN2BAS* loci was evaluated under conditions of JUN-KD or Ctrl siRNA transfection. **g** Serial ChIP-qPCR with JUN first IP followed by p300 second IP. **h** Serial ChIP-qPCR with p300 first IP followed by JUN second IP (mean ± SD, *n* = 3)

In our previous studies, we identified enhancer regions associated with *SMAD3* and *CDKN2BAS* genes [33]. The presumptive causal variants located in these regions, rs17293632 in the *SMAD3* enhancer and rs1537373 in the *CDKN2BAS* enhancer, are in close proximity to AP1 and TCF21 binding sites [2–4, 33, 35, 46, 47]. These variants are located in HCASMC ATACseq open chromatin and, by eQTL and allele-specific expression, have been linked to the expression of *SMAD3*, *CDKN2BAS*, or *CDKN2B* [33, 34]. Thus, we designed two ChIP-qPCR primer sets for the *SMAD3* enhancer. One set of primers amplifies the *SMAD3*-*1* region, containing the AP-1 binding motif which is disrupted by rs17293632, and the second set amplifies the *SMAD3*-*2* region containing the TCF21 binding motif which is + 505 bp downstream from AP-1 binding site (Additional file 1: Figure S1D). However, AP-1 factors can also bind to the *SMAD3-2* region to some degree as shown by enrichment for JUN ChIPseq reads (Additional file 1: Figure S1D), and although not evident with the genome browser settings shown, TCF21 can bind to *SMAD3-1* with a small but significant number of TCF21 ChIPseq reads (Additional file 1: Figure S1D). Another primer set was designed to amplify the *CDKN2BAS-CDKN2B* enhancer, containing both AP-1 and TCF21 binding motifs downstream from rs1537373 (Additional file 1: Figure S1E). To better understand the mechanism of how AP-1 affects the transcription of CAD genes, we evaluated transcription factor binding and chromatin state in these loci in HCASMC by ChIP-qPCR. Compared with control siRNA transfection, *JUN* knockdown resulted in significantly decreased JUN binding in both *SMAD3* (*SMAD3-1* and *SMAD3-2*) and *CDKN2BAS* loci (Fig. 2a). Since JUN promotes the endogenous expression of *TCF21* [20], we overexpressed *TCF21* with lentivirus transduction to maintain the *TCF21* expression level in *JUN* knockdown and control cells (Fig. 2b, Additional file 1: Figure S2A, S2B). With sustained TCF21 level in these cells, JUN knockdown still resulted in reduced TCF21 binding to these loci (Fig. 2c, Additional file 1: Figure S2A, S2B). Associated with JUN’s decrease, histone H3K27 acetylation was also decreased (Fig. 2d). Lower H3K27ac levels resulted in decreased chromatin accessibility in these loci (Fig. 2e). These data demonstrate that JUN binding in these candidate causal CAD loci leads to altered histone modification, chromatin accessibility, and recruitment of chromatin remodeling factors, as a potential mechanism of their effects on gene expression in HCASMC.

### JUN recruits EP300 to acetylate H3K27 and open chromatin in CAD loci

We next investigated how AP-1 complex transcription factors regulate histone modification and chromatin state. In vitro purification studies have provided evidence that the AP-1 complex interacts with histone acetyltransferases (HATs), suggesting that EP300 (p300) may be involved in transcriptional regulation in HCASMC [48–50]. To test this hypothesis, we assessed whether p300 binding in *SMAD3* and *CDKN2BAS* loci is dependent on JUN expression level. ChIP-qPCR studies showed p300 binding requires the presence of JUN as p300 binding is significantly decreased in the absence of JUN (Fig. 2f). Consistent with p300’s role as a HAT, JUN overexpression increased H3K27ac level in the *SMAD3* locus, which could be negated by knocking down p300 (Additional file 1: Figure S2C, S2D). These studies demonstrated that JUN regulates H3K27ac of enhancers in a p300-dependent manner. Interestingly, although JUN effects on TCF21 binding can also be reversed by *p300* knockdown, *p300* knockdown alone cannot affect the TCF21 binding in the *SMAD3* locus (Additional file 1: Figure S2E). These findings suggest that it is AP-1, not the histone modification enzymes, that plays a leading role in guiding the binding of other TFs, such as TCF21.

To further explore the relationship between AP-1 and HATs, we employed serial ChIP to study their interactions. With the first immunoprecipitation (IP) using JUN antibody, followed by a second IP using p300 antibody with the eluate from the first IP, we observed significant enrichment of both JUN and p300 on *SMAD3-1* and *CDKN2BAS* loci, while the *SMAD3-2* locus showed enrichment for JUN and modest enrichment for p300 that was not significant (Fig. 2g). With the opposite direction serial ChIP, we also observed similar enrichment of p300 and JUN at *SMAD3-1* and *CDKN2BAS* loci (Fig. 2h). Together, these data suggest that p300 is recruited by AP-1 to the CAD *SMAD3* and *CDKN2BAS* loci, thereby facilitating JUN-mediated histone acetylation, chromatin accessibility, and binding of other transcription factors.

### TCF21 reduces H3K27ac level and chromatin accessibility but not JUN binding at CAD loci

**Fig. 3:**
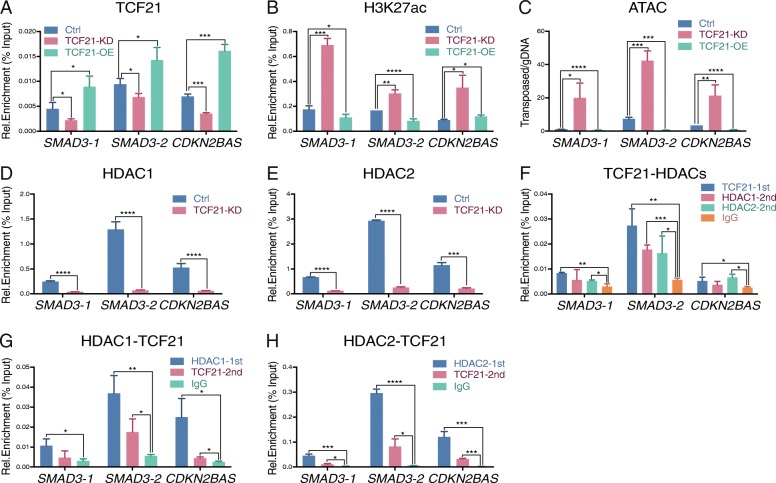
TCF21 recruits HDACs 1 and 2, promotes deacetylation at H3K27ac, and decreases chromatin accessibility. All panels show ChIP-qPCR at *SMAD3-1, SMAD3-2*, and *CDKN2BAS* locus regions with input normalization in HCASMC. **a** TCF21 binding, **b** H3K27ac level, and **c** chromatin accessibility (ATAC-PCR) were evaluated under the conditions of *TCF21* (TCF21-KD) or scrambled (Ctrl) siRNA transfections or lentivirus overexpression of *TCF21* (TCF21-OE). **d** HDAC1 and **e** HDAC2 binding were evaluated by ChIP-qPCR with TCF21-KD. **f** Serial ChIP-qPCR with TCF21 first IP followed by HDAC1 or HDAC2 second IP. **g** Serial ChIP-qPCR with HDAC1 first IP followed by TCF21 second IP. **h** Serial ChIP-qPCR with HDAC2 first IP followed by TCF21 second IP (mean ± SD, *n* = 3)

In contrast to AP-1, TCF21 appears to function primarily as a transcriptional repressor [22, 23], suggesting that its effect on transcription in CAD loci might be quite different from AP-1. This is consistent with other bHLH factors that oppose AP-1 actions [51]. To better understand how TCF21 modulates gene expression, we investigated the level of H3K27ac, chromatin accessibility, and JUN binding in the *SMAD3* and *CDKN2BAS* loci under *TCF21* siRNA knockdown or lentivirus overexpression in HCASMC. The ChIP-qPCR data from cells with *TCF21* knockdown show that with decreased TCF21 binding in these loci (Fig. 3a), the H3K27ac levels and chromatin accessibility are increased (Fig. 3b, c). Decreased chromatin accessibility is observed at *SMAD3-1* and *SMAD3-2* with increased *TCF21* expression which is consistent with the observed effects on H3K27ac status, although *CADKN2BAS* shows a slight and unexpected increase in H3K27ac when TCF21 binding is elevated by lentivirus (Fig. 3a–c). Interestingly, JUN binding in these loci is not significantly affected by reduced or elevated TCF21 binding (Additional file 1: Figure S3A). Combined with the *JUN* knockdown data, these findings suggest that JUN binding is upstream and likely precedes TCF21 binding, with the AP-1 complex serving as a pioneer factor that recruits TCF21 itself after binding DNA and modifying the chromatin environment, as has been characterized in the case of the glucocorticoid receptor [30]. These data are consistent with our previous observations that JUN activation precedes *TCF21* expression [20]. Together, these results indicate that TCF21 is a suppressor of the two CAD presumptive causal genes under study here, *SMAD3* and *CDKN2BAS*, and suggest that the transcriptional inhibition by TCF21 is possibly mediated through H3K27ac, but that this effect does not alter AP-1 binding.

### TCF21 recruits HDAC1 and 2 to deacetylate H3K27 at CAD loci

It has been previously shown that TCF21 function in the androgen receptor pathway is histone deacetylase (HDAC) 1 dependent [52] and that it directly associates with HDAC2 [53], suggesting a possible mechanism for how TCF21 suppresses gene transcription in HCASMC. To answer this question, we evaluated both HDAC1 and HDAC2 binding in *SMAD3* and *CDKN2BAS* loci in ChIP-qPCR experiments. These data showed a significant reduction in both HDAC1 and HDAC2 binding under *TCF21* siRNA knockdown (Fig. 3d, e), along with decreased TCF21 binding level (Additional file 1: Figure S3B).

We further investigated the interaction between TCF21 and HDACs with serial ChIP experiments. With the first IP using TCF21 antibody, followed by the second IP using HDAC1 or HDAC2 antibodies from the elution of the first IP, we observed significant enrichment of both HDAC1 and HDAC2 at *SMAD3* and *CDKN2BAS* loci in HCASMC (Fig. 3f). In a second series of experiments, we performed serial ChIP in the reverse direction, first IP with HDAC1 or HDAC2 antibody and second IP with TCF21 (Fig. 3g, h). These data suggest that TCF21 recruits HDAC1 and HDAC2 to *SMAD3* and *CDKN2BAS* genetic loci to deacetylate H3K27 and thereby suppress transcription.

### JUN and TCF21 co-localize at CAD enhancers and directly interact through protein-protein binding

**Fig. 4:**
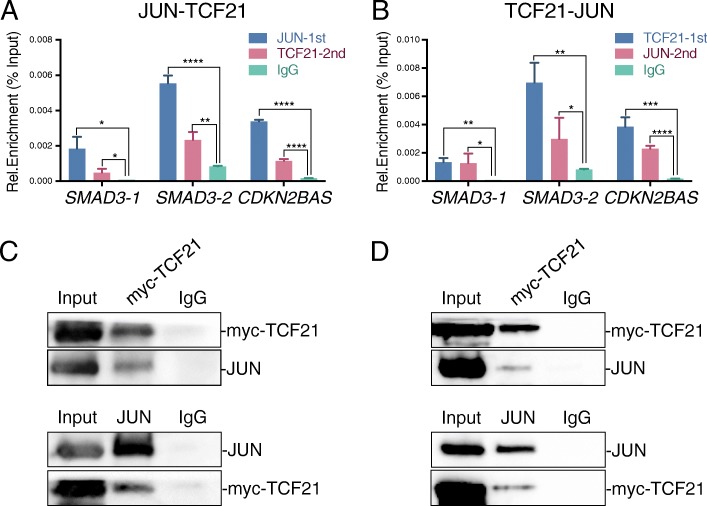
JUN and TCF21 co-occupy chromatin at *SMAD3* and *CDKN2BAS* loci. **a** Serial ChIP-qPCR with JUN first IP followed by TCF21 second IP. **b** Serial ChIP-qPCR with TCF21 first IP followed by JUN second IP (mean ± SD, *n* = 3). **c** Interaction between TCF21 and JUN on chromatin was evaluated by ChIP-western blotting, IP with myc-TCF21 (top) followed by JUN western, or with reverse conditions (bottom). Myc-tagged TCF21 and non-tagged JUN expression constructs were transfected into HEK293 cells. **d** Co-immunoprecipitation of TCF21 and JUN with IP of myc-TCF21 followed by JUN western (top) and reverse conditions (bottom). Myc-tagged TCF21 and non-tagged JUN were transfected into HEK293 cells

AP-1 and TCF21 binding sites are juxtaposed in the *SMAD3* and *CDKN2BAS* loci (Additional file 1: Figure S1D, S1E), suggesting a possible direct interaction between these two factors. We employed serial ChIP, ChIP-western blotting, and co-IP experiments to investigate this hypothesis. First, serial ChIP studies provided evidence for chromatin-dependent interaction at the *SMAD3* and *CDKN2BAS* loci. Either with the first IP using a JUN antibody, followed by a second IP using TCF21 antibody with the eluate of the first IP (Fig. 4a), or performing the IPs in the opposite direction (Fig. 4b), co-enrichment of TCF21 and JUN were detected at the *SMAD3* and *CDKN2BAS* loci in HCASMC. Second, we performed ChIP-western blotting experiments with formaldehyde crosslinked HEK293 cell line transfected with plasmids encoding myc-tagged TCF21 and native JUN. IP was performed with myc-tag or JUN antibodies in the ChIP buffers followed by reversal of crosslinking and western blotting (Fig. 4c). These data, from IP performed in both directions, provide additional evidence for co-localization of AP1 and TCF21 in enhancer regions of these two CAD loci. Finally, we transfected *TCF21* and *JUN* plasmids into HEK293 cells and performed direct co-IP experiments to look for protein-protein interaction of these factors. The results showed that JUN can be pulled down with myc-tagged TCF21 IP, and myc-tagged TCF21 also identified in JUN IP (Fig. 4d). Taken together, these data provide compelling support for AP-1 and *TCF21* binding together at sites in the genome and directly interacting via protein-protein binding.

### JUN and TCF21 binding is co-localized genome-wide and these factors regulate H3K27ac levels in the shared loci

We have previously reported ChIPseq data for TCF21 and JUN in HCASMC [23] and documented a significant overlap of the binding regions in CAD-associated loci [33]. To further investigate the co-localization of JUN and TCF21 binding genome-wide, and the resulting impact of their binding on local epigenomic features, we have mapped H3K27ac histone modifications in conjunction with JUN and TCF21 ChIPseq under knockdown and control conditions in HCASMC.

**Fig. 5:**
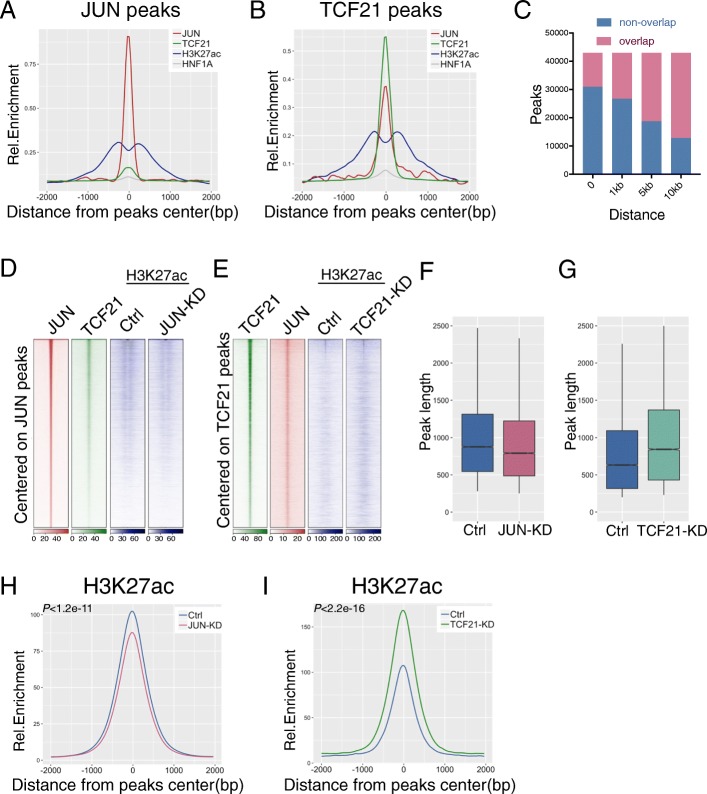
JUN and TCF21 co-localize genome-wide and regulate H3K27ac chromatin modification. **a** JUN ChIPseq peaks and **b** TCF21 peaks were extended +/− 2 kb from summit, and a density plot created for RPM normalized enrichment levels of JUN, TCF21, and H3K27ac ChIPseq in HACSMC, along with control transcription factor HNF1A. **c** The number of TCF21 peaks overlapped with JUN binding for distances between peaks less than 1 kb, 5 kb, and 10 kb. **d** Heatmap distribution of ChIPseq data for JUN, TCF21, and H3K27ac with control (Ctrl) or *JUN* knockdown (JUN-KD) centered on JUN peaks, or **e** *TCF21* knockdown (TCF21-KD) centered on TCF21 peaks within a 4-kb window. **f** Peak length distribution of Ctrl or JUN*-*KD, and **g** Ctrl or TCF21-KD H3K27ac ChIPseq in HCASMC (box plots with *Wilcoxon tests*, *P* < 2.2e−16). **h** H3K27ac ChIPseq enrichment level of Ctrl or JUN-KD, and **i** Ctrl or TCF21-KD, centered on all H3K27ac peaks with +/− 2 kb extension, normalized by RPM

First, we compared the binding of native levels of JUN and TCF21, along with H3K27ac modifications, in the control ChIPseq datasets. We found co-localization of TCF21 and H3K27ac enrichment centered on total 85,695 JUN peaks, compared with the negative control TF HNF1A. Visualization of this co-localization with a density plot for normalized ChIPseq peaks showed H3K27ac modification overlapping and flanking the JUN peaks, along with TCF21 overlap as previously described (Fig. 5a) [33]. Focusing on a total 42,490 TCF21 peaks revealed co-localization of JUN binding and H3K27ac marked regions. A density plot showed more prominent overlap with JUN binding and H3K27ac mapping with a similar pattern of co-localization (Fig. 5b). We also plotted position weight matrix (PWM) scans for predicted binding at JUN and TCF21 peaks, which showed similar co-localization of the TFs (Additional file 1: Figure S4A, S4B). To quantify the number of overlapping JUN and TCF21 peaks, we intersected these two groups of ChIPseq peaks, identifying 12,033 TCF21 peaks that directly overlap with JUN peaks (*P* < 1.41e−91, Fig. 5c). The overlapped TCF21 peak number increased rapidly with the distance from JUN, and more than 70% of TCF21 peaks (30,120 out of 42,490) are found less than 10 kb from JUN binding sites (Fig. 5c), suggesting relative proximity of their binding genome-wide and possible regulatory interactions.

Co-localization of binding was verified by investigating the binding site distance from the control factor HNF1A, a TF that is functional primarily in the liver, and has different binding features from TCF21 and AP-1. We intersected DNaseI open chromatin data for HepG2 liver cells from ENCODE and our ATACseq data for HCASMC to get the common open chromatin regions in these two cell types, and analyzed the closest distances of binding sites for these various factors. The histogram of distance shows no significant difference between the spacing of TCF21 from JUN, JUN from TCF21, or TCF21 from HNF1A in the common open chromatin regions (Additional file 1: Figure S4D). However, if we exclude the common open regions of ENCODE from HCASMC open regions to obtain HCASMC-specific open chromatin regions, the median distance between binding sites of TCF21 and JUN is 12,781 bp, while that of TCF21 and HNF1A is 78,559 bp (Additional file 1: Figure S4E), suggesting that the co-localization of TCF21 and JUN is smooth muscle cell type-specific.

Since we have shown that AP-1 and TCF21 can both modulate H3K27ac histone modification in CAD loci in HCASMC, we were interested in mapping and comparing loci across the genome where these TFs regulate H3K27ac status in HCASMC. Thus, we performed H3K27ac ChIPseq under *TCF21* shRNA or *JUN* siRNA knockdown, obtaining 24,065 and 24,883 total peaks under these conditions, respectively. Heatmap distributions of JUN peaks showed genome-wide enrichment of TCF21 binding, with enrichment of H3K27ac showing a flanking pattern (Fig. 5d) as in the density plot, and diminished signal in the *JUN* knockdown condition. Similar heatmaps of TCF21 binding showed enrichment for JUN co-localization, with more widely distributed H3K27ac enrichment that is more pronounced with *TCF21* knockdown (Fig. 5e). To quantify the change in H3K27ac status genome-wide, we evaluated the average enrichment levels on H3K27ac peaks found in the sequencing data. Compared with scrambled control, H3K27ac level in *JUN* siRNA knockdown showed a change in peak length distribution with shorter length peaks with *JUN* knockdown and longer peaks with *TCF21* knockdown (Fig. 5f, g) (*Wilcoxon tests*, both *P* < 2.2e−16).). In addition, *JUN* siRNA knockdown showed a modest but significant reduction in H3K27ac peak height for all peaks (Fig. 5h), while the level in *TCF21* shRNA knockdown showed a significant increase (Fig. 5I).

Further, we identified loci where H3K27ac is regulated by both JUN and TCF21 in HCASMC. With *JUN* knockdown, we obtained 10,882 decreased and only 391 increased H3K27ac peaks compared with control. On the other hand, 42 decreased and 14,234 increased H3K27ac peaks were found in *TCF21* knockdown. With a more stringent *q* value cutoff (*q* < 1e−10 for TCF21 and *q* < 1e−5 for JUN), chosen to give equivalent numbers of peaks, we found 7017 peaks with H3K27ac increased with JUN knockdown and 6026 peaks with H3K27ac decreased with TCF21 knockdown. Out of these, 3093 peaks were directly overlapping (Additional file 1: Figure S4F). We also intersected those 3093 overlapping H3K27ac peaks with TCF21 and JUN ChIPseq peaks, showing that 749 peaks overlap with TCF21, 699 with JUN, and 224 with both (Additional file 1: Figure S4G). Therefore, 1124 H3K27ac peaks overlap with at least one of the two transcription factor ChIPseq sites, suggesting that more than one third of these sites are directly regulated by TCF21 and JUN, while two thirds are indirectly regulated by TF downstream of TCF21 and JUN. These data indicate that H3K27ac level is regulated at the genome-wide level by AP-1 and TCF21, thus supporting our ChIP-qPCR data and suggesting a genome-wide epigenomic interaction of these factors. Overall, changes in H3K27ac level with *JUN* knockdown were not as pronounced as those seen for *TCF21* knockdown, possibly due to the fact that there are a number of bZip factors that can bind and regulate H3K27ac at AP-1 sites in addition to JUN, leading to a smaller effect size.

Finally, we employed Genomic Regions Enrichment of Annotations Tool (GREAT) to assign the common 3093 H3K27ac regions to genes, and this collection of 2580 target genes were used in gene ontology analysis with DAVID. Significant terms identified by the Biological Process analysis included VEGF, TGFβ and EGF signaling pathways, cell adhesion, migration, and proliferation, which are highly relevant to smooth muscle cellular functions (Additional file 1: Figure S4H, Additional file 2: Table S1). Significant KEGG terms included VEGF, TGFβ and Hippo pathways, focal adhesion, and cell cycle (Additional file 1: Figure S4I, Additional file 2: Table S1). These data suggest that AP-1- and TCF21-regulated H3K27ac loci are highly associated with basic HCASMC functions that are relevant to CAD pathophysiology.

### JUN and TCF21 regulate chromatin accessibility in HCASMC

**Fig. 6:**
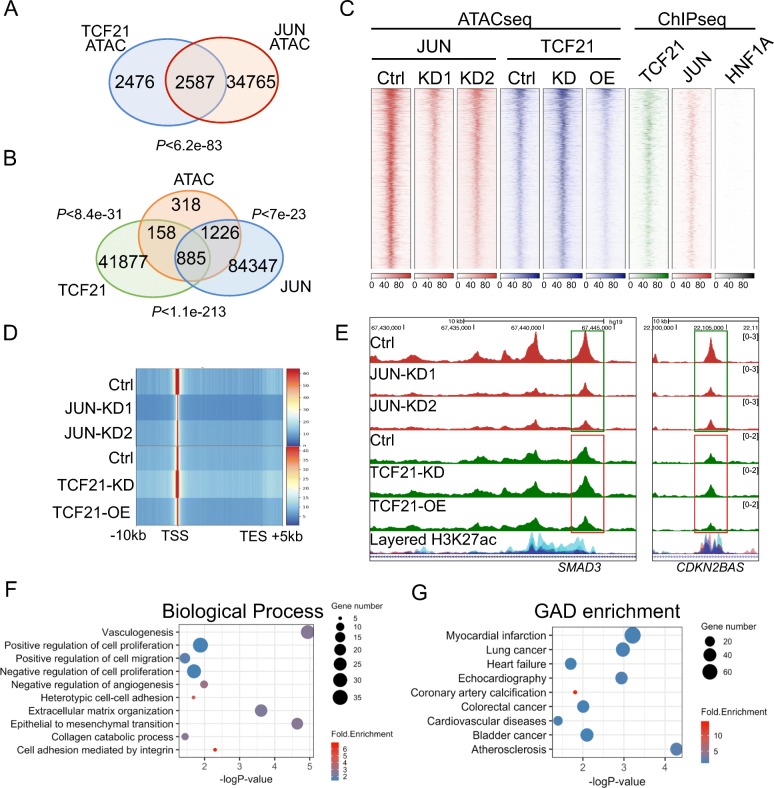
JUN and TCF21 regulate chromatin accessibility at CAD loci. **a** Venn diagram showing the number of overlaps between TCF21-regulated (intersection of *TCF21* knockdown upregulated and overexpression downregulated ATAC peaks, *q* < 0.01) and JUN-regulated (intersection of two *JUN* knockdown downregulated ATAC peaks, *q* < 1e−10 and fold change < 0.5) open chromatin regions in HCASMC. **b** Venn diagram showing the number of overlaps between JUN plus TCF21-regulated open chromatin regions (from **a**) and TCF21 or JUN peaks (*q* < 0.01) in HCASMC. **g** Heatmap distribution of ATACseq for control (Ctrl) or two *JUN* knockdowns (KD1 and KD2), ATACseq of control (Ctrl) or *TCF21* knockdown (TCF21-KD) or *TCF21* overexpression (TCF21-OE), and ChIPseq of JUN and TCF21, all centered on JUN plus TCF21-regulated open chromatin regions within a 4-kb window in HCASMC. HNF1A serves as a control transcription factor. **d** Heatmap distribution of ATACseq peaks for Ctrl, JUN-KD1, or JUN-KD2, and ATACseq of Ctrl, TCF21-KD, or TCF21-OE on genes within 10 kb upstream and 5 kb downstream of the transcription start site, in loci located in JUN plus TCF21-regulated open chromatin regions. **e** Pattern of ATACseq mapping of open chromatin at the human *SMAD3* and *CDKN2BAS* loci, with *JUN* knockdown (JUN-KD1, -KD2), *TCF21* knockdown (TCF21-KD), or *TCF21* overexpression (TCF21-OE). ENCODE-layered H3K27ac data are also shown. **f** Biological processes and **g** GAD disease enrichment from DAVID Gene Ontology analysis of genes located in JUN plus TCF21-regulated open chromatin regions. Genes were assigned by GREAT with “single nearest” mode

Open chromatin regions in HCASMC are enriched for CAD-associated loci and GWAS variants [19, 33]. To investigate the epigenetic implications of these observations, we employed ATACseq to generate epigenomic profiles in HCASMC with *JUN* knockdown, *TCF21* knockdown, and *TCF21* overexpression. With *TCF21* knockdown, we obtained 54,298 increased open chromatin regions and 2218 decreased regions; while 9932 decreased regions were found with *TCF21* overexpression, all with *q* < 0.01. Since TCF21 is mainly identified as a transcriptional suppressor [22, 44], we intersected the 54,298 increased regions with knockdown and the 9932 decreased regions with overexpression, identifying 5063 overlapped high confidence TCF21 targets. Similarly, we intersected 110,373 and 109,143 decreased regions identified with *JUN* knockdown by two different shRNAs, sh*JUN*-1 and -2, thus identifying 37,352 JUN high confidence ATACseq targets with *q* < 1e−10 and fold change < 0.5. There were 2587 overlaps within the TCF21 and JUN target regions, which we identified as loci regulated by the two TFs with high confidence (*P* < 6.2e−83, Fig. 6a). We also integrated these loci with ChIPseq profiles for JUN and TCF21 binding as well as those for H3K27ac modifications. A large portion of the 2587 JUN and TCF21 ATACseq targets overlap with TCF21 and JUN peaks; 1043 with TCF21, 2111 with JUN, and 885 with both (Fig. 6b). The H3K27ac peaks were also highly enriched in the open chromatin regions of *JUN* or *TCF21* knockdown, with ~ 90% overlap with ATAC peaks (Additional file 1: Figure S5A, S5B). Further, we centered the 2587 loci for heatmap clustering, showing the co-localization of these open chromatin regions with JUN and TCF21 binding, but not with the negative control HNF1A (Fig. 6c). The enrichment levels of ATACseq on these loci are reduced in *JUN* knockdown while they are upregulated in *TCF21* knockdown and downregulated in *TCF21* overexpression (Fig. 6c), which is consistent with the regulated peak numbers above. We also discovered several AP-1 and TCF21/TCF12 binding motifs using the HOMER “*known*” motif analysis on these 2587 loci with low *P* values (Additional file 1: Figure S5C). These analyses provide a map of genomic regions targeted by AP-1 and TCF21 in HCAMSC.

To investigate the genes in the open chromatin regions where JUN and TCF21 are co-localized, we assigned those loci to genes using GREAT. At the identified 936 genes, chromatin accessibility is downregulated by TCF21 and upregulated by JUN, especially around the TSS (Fig. 6d). At the *SMAD3* and *CDKN2BAS* enhancers studied above, we observed pronounced chromatin accessibility differences with *JUN* or *TCF21* disruptions (Fig. 6e). In addition, smooth muscle cell marker genes such as *ACTA2* and *MYH11* were regulated with similar directionality (Additional file 1: Figure S5D). At the genome level, GO analysis identified a large number of the 936 genes to be significantly enriched in smooth muscle cell functions and CAD-associated terms, including vasculogenesis, angiogenesis, cell adhesion, and proliferation in biological processes; myocardial infarction, cardiovascular diseases, and coronary artery calcification in GAD enrichment; ECM and cell adhesion in KEGG pathways (Fig. 6f, g, Additional file 1: Figure S5E, Additional file 3: Table S2, Additional file 4: Table S3). The change in chromatin accessibility results in differential expression levels of those genes, ~ 2/3 of which are upregulated in mRNA level with *TCF21* siRNA knockdown (Additional file 1: Figure S5F, Additional file 4: Table S3). In summary, these data reveal that AP-1 and TCF21 regulate chromatin modification and accessibility genome-wide in loci that regulate fundamental SMC processes; some of which are important mediators of CAD risk.

### CAD-associated variants are enriched in open chromatin regions regulated by JUN and TCF21

Given that the variation in chromatin accessibility and TF binding is a dominant mechanism of variation in gene expression, we were interested to identify and experimentally validate variants located in the 2587 regions of the genome that are regulated by AP-1 and TCF21 in HCASMC (Fig. 6a). First, we intersected those open chromatin regions with the GTEx Coronary Artery (CA) eQTL public variant database [54], as well as eQTLs we have mapped in HCASMC (*q* < 0.05) [19], and CARDIoGRAMplusC4D CAD [43]-associated SNPs (*P* < 0.05). There were 491 GTEx CA and 643 HCASMC eQTLs, and 857 CARDIoGRAMplusC4D SNPs located in these loci (Additional file 1: Figure S6A) [2, 4, 19, 55]. In particular, 643 HCASMC eQTLs were associated with 193 genes, which were enriched in CAD-associated terms in GO analysis [19]. Identified terms are quite similar to those found in AP-1- and TCF21-targeted loci (Additional file 1: Figure S6B, C, D, Additional file 5: Table S4). The expression level of these genes was also investigated in a previous *TCF21* knockdown RNAseq study [22], showing that > 70% differentially expressed genes are elevated at the transcriptional level (Additional file 1: Figure S6E). With a second approach, employing the GWAS catalog with CARADIoGRAM+C4D data added, we intersected all GWAS variants within AP-1 plus TCF21-targeted regions of open chromatin; the results show that significant (low *P* values) and highly enriched (high fold change) GWAS CAD SNPs were found in those regions (Additional file 1: Figure S6F). Further, we overlapped the JUN and TCF21 intersected peaks, (Additional file 1: Figure S6G), total TCF21 peaks (Additional file 1: Figure S6H), and total JUN peaks (Additional file 1: Figure S6I) with GWAS catalog SNPs. CAD-associated terms were highly enriched in overlapped regions of the two ChIPseq datasets compared to the individual datasets. Terms “CARDIoGRAM+C4D,” “Coronary Artery Calcification,” and “Coronary Artery” showed a 3–6-fold enrichment in the overlapping data analysis while JUN, and TCF21 alone had only 1–3-fold enrichment.

### Allele-specific binding of JUN and TCF21 leads to a transcriptional imbalance of presumptive CAD causal genes

**Fig. 7:**
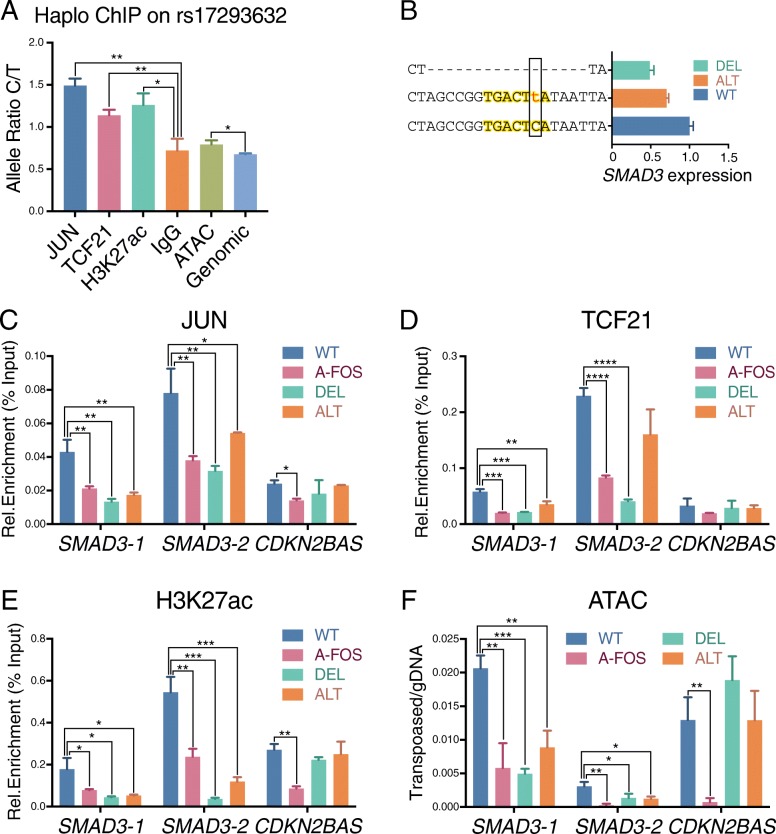
Allele-specific binding of JUN, TCF21, and H3K27ac enrichment at the *SMAD3* locus. **a** HaploChIP of JUN, TCF21, H3K27ac, and ATAC at rs17293632 was evaluated by qPCR with allele-specific TaqMan probes in heterozygous HCASMCs. Enrichment was normalized with input, and data was shown as C/T ratio. **b** *SMAD3* expression levels in wild type (WT), deletion (DEL), or converted (ALT) HEK293 CRISPR/Cas9 edited cell lines as detected by qPCR. The genomic sequence of CRISPR edits are shown for corresponding cell lines. **c** ChIP-qPCR of JUN, **d** TCF21, **e** H3K27ac, and **f** ATAC-qPCR showing the differential transcription factor binding, H3K27ac modification, or ATACseq open chromatin at *SMAD3-1*, *SMAD3-*2, and *CDKN2BAS* locus regions for WT or CRISPR HEK293 cell lines. Dominant negative A-FOS was expressed as a positive control of JUN inhibition, and all data normalized versus input (mean ± SD, *n* = 3)

With these data regarding the role of AP-1 and TCF21 in genome-wide regulation of epigenetic features in CAD loci, we wanted to investigate the effect that such features have on allele-specific TF binding and CAD gene expression. In these experiments, we employed HCASMC from individuals heterozygous for candidate CAD regulatory SNP rs17293632 at *SMAD3* that localize to a validated canonical AP-1 site in this gene [33]. The native C allele that is consistent with AP-1 factor binding was found to be associated with allelic expression imbalance of *SMAD3* as detected by qRT-PCR, along with evidence of allelic differences in chromatin accessibility as reflected by ATACseq read counts, allele-specific binding of JUN, TCF21, and H3K27ac as identified with haploChIP studies (Fig. 7a).

To more formally address this question, we conducted follow-up studies in cells with CRISPR/Cas9 genome editing of the SNP rs17293632 in HEK293 cells. A series of edited lines were identified by genomic sequencing. A motif deleted (DEL) line and a single base-pair alteration line (ALT) which edited the C to T allele showed decreased SMAD3 expression compared to the native allele (Fig. 7b). These lines were used in ChIP-qPCR experiments which showed that motif deletion causes a significant reduction of JUN binding at rs17293632, as did the single nucleotide alteration (Fig. 7c). The decreased JUN binding can be observed at the edited rs17293632 loci but not with the native rs1537373 *CDKN2BAS* variant. The JUN-dominant negative factor A-FOS can reduce the binding on both loci, showing that the reduction of JUN binding is caused by site-specific genome editing (Fig. 7c). We also investigated TCF21 binding and the chromatin state at these loci. Since no endogenous *TCF21* expression was detected in HEK293 cells, we overexpressed *TCF21* in these lines using lentivirus transduction. As expected, decreased JUN binding level resulted in reduced TCF21 binding, H3K27ac level, and chromatin accessibility at rs17293632 but not at rs1537373 (Fig. 7d–f). These data demonstrate that CAD variants can lead to allele-specific TF binding and imbalanced expression level of regulated genes.

## Discussion

**Fig. 8:**
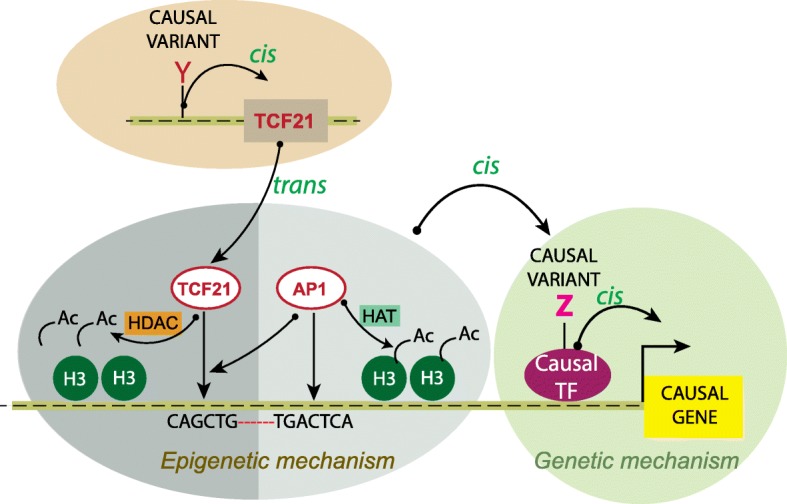
Mechanism of TCF21 and AP-1 epigenetic interactions in the context of CAD-associated genetic loci. TCF21 is a bHLH transcription factor associated with CAD as depicted here due to allelic variation in causal variants (Y), and its transcriptional regulatory function accounts for the attributable genetic risk at the 6q23.2 locus (brown oval). TCF21 binding is enriched in other CAD loci, where it interacts with AP-1 factors (JUN) that co-localize at these sites (gray oval). JUN promotes recruitment of HAT p300 to promote H3K27ac histone acetylation and open chromatin to recruit TFs, including TCF21, which in turn recruits HDACs 1 and 2 that function to oppose AP-1 effects. These epigenetic effects contribute in *cis* to the regulation of expression of the causal gene through alteration in the binding of the causal TF through CAD-associated variant Z (green circle) or other mechanisms of disease at this locus. Such interactions likely contribute to attributable genetic risk at both the TCF21 and downstream loci

In conjunction with previously published data, the data presented here indicate that there are multiple mechanisms by which AP-1 factors may regulate transcription in CAD-associated loci and thus a disease risk. First, there are now several examples of AP-1 binding sites which are altered by causal variation, leading to differences in gene expression. An AP-1 cognate binding site in an intron of the *SMAD3* gene has been shown to regulate expression of this gene and implicated as causal for both CAD and autoimmune diseases [20, 32, 34, 35, 56]. Also, causal variation at two CAD-associated alleles of *TCF21* disrupts canonical AP-1 binding sites, which binds AP-1 factors and promotes expression of *TCF21* [20]. A second mechanism, delineated by work presented here, provides compelling evidence that AP-1 may regulate expression of nearby TFs or chromatin regulatory features that are causal in disease loci by altering nearby epigenetic features and chromatin accessibility, i.e., through pioneer epigenetic functions (Fig. 8). This mechanism can also be extended to include the situation presented here whereby AP-1 regulates the binding and function of another functional TF such as TCF21, that in turn regulates the causal transcriptional mechanism. Finally, AP-1 factors are well known to interact with bHLH factors such as MYOD [57], and this fact in conjunction with evidence presented here for TCF21-AP-1 interaction suggests that AP-1 may modulate binding of other TFs through direct protein-protein interaction.

These data add to our understanding of how TCF21 affects CAD gene expression and function. TCF21 has a developmental role in coronary smooth muscle, specifically suppressing the expression of SMC lineage markers through transcriptional regulation/suppression (e.g., ACTA2) and possibly affecting the myocardin-SRF complex which serves as a master regulator in this cell type. Loss of *TCF21* leads to increased SMC differentiation of CASMC. Reactivation of *TCF21* may have a similar role in SMC in disease, promoting dedifferentiation and migration, which is protective in this setting, based on directionality from human genetics studies [21, 22]. In addition to its capacity to regulate the transcriptional effect of the causal variant in the CAD locus, these data show that TCF21 has another mechanism of effect, which works through local epigenetic modification to regulate access of TFs and chromatin regulators to modulate gene expression. Further, these studies suggest that TCF21 can serve as a lineage partner for AP-1, similar to what has been shown for AP-1 interactions with PU.1 in macrophages, and ETS and GATA factors in endothelial cells where AP-1 promotes cell-restricted enhancer function [28, 58].

One important question that arises from these studies is the nature of the TCF21-AP-1 interaction in terms of CAD gene expression and disease risk. AP-1 is activated and binds to the *SMAD3* and *CDKN2BAS* CAD loci, recruiting HAT activity and thus promoting an open chromatin configuration (Fig. 8). This promotes TCF21 binding, which in turn recruits HDACs, thus setting up a competition for the status of local chromatin configuration. The best explanation for these findings is that they represent a counter-regulatory pathway, with independent upstream epigenetic pathways regulating one or the other of these factors, and overall disease versus risk being determined by the cellular milieu in the vascular wall. It is important to note that the balance of regulation of promotion versus inhibition of transcription is more complex than just TCF21-AP-1 interactions, as these factors also likely regulate binding of CTCF, CEBP, and other TFs that bind in this region through their epigenetic effects. Importantly, for the *SMAD3* and *CDKN2BAS* presumptive causal genes, TCF21 inhibits disease-promoting effects of AP-1 on the risk alleles at these two loci [19, 33]. The same type of relationship has been characterized at CAD loci where TCF21 interacts with SMAD3, with the binding of both of these factors co-localizing genome-wide in a number of CAD loci [37]. That interaction is also primarily competitive, with TCF21 blocking SMAD3 binding through epigenetic modulation. The effects of *SMAD3* expression are opposite to *TCF21* and promote disease risk, whereas AP-1 can be both protective through the promotion of TCF21 expression, and risk promoting through increasing *SMAD3* expression [37].

While these studies highlight the interaction of AP-1 factors with TCF21 in loci that are linked to CAD, they likely represent a mechanism that is applicable to other human diseases. For instance, the mapped loci showing co-localization of AP-1 and TCF21 are enriched for genes that contribute to *VEGF*, *TGFβ*, *EGF*, Hippo, focal adhesion, and cell cycle biological processes (Additional file 1: Figure S4H). These terms are well known to associate with various forms of cancer, and the data presented here are consistent with both the involvement of AP-1 and TCF21 interactions in the biology of the tumor cell, since TCF21 is a well-known tumor suppressor [59], as well as the angiogenic processes that support tumor growth and expansion [60, 61].

## Conclusions

These data characterizing the TCF21-AP-1 counter-regulatory pathway in CAD loci suggests that the epigenetic landscape in disease loci is more complex than previously thought and that part of the attributable risk for CAD-associated transcription factors such as TCF21 may be due in part to their functions in *trans*, mediated by epigenetic effects at other CAD loci across the genome. The risk that resides in each disease locus would thus be a combination of the local mechanism involving the causal allele and gene, as well as that due to modulation of the epigenome by TFs such as TCF21 (Fig. 8).

## Additional files

Additional file 1: Supplemental figures and related figure legends. (PDF 2496 kb)

Additional file 2: **Table S1.** Biological processes and KEGG pathways from DAVID Gene Ontology analysis of JUN-TCF21 regulated H3K27ac genes. (XLSX 16 kb)

Additional file 3: **Table S2.** GO analysis of genes in or nearby the open chromatin regions where JUN and TCF21 are co-localized. (XLSX 15 kb)

Additional file 4: **Table S3.** Genes in or nearby the open chromatin regions where JUN and TCF21 are co-localized, genes assigned by GREAT. (XLSX 22 kb)

Additional file 5: **Table S4.** Biological processes, KEGG pathways, and GAD disease enrichment of DAVID Gene Ontology analysis of HCAMSC eQTL target genes located in JUN plus TCF21 regulated open chromatin regions. (XLSX 14 kb)

## Abbreviations

AP-1: Activated protein 1
CAD: Coronary artery disease
*CDKN2B-AS1*: *CDKN2BAS*
eQTL: Expression quantitative trait locus
GWAS: Genome-wide association studies
HAT: Histone acetyltransferase
HCASMC: Human coronary artery smooth muscle cells
HDAC: Histone deacetylase
SMC: Smooth muscle cells
TF: Transcription factor
