# Supplementary material for: TCF21 and AP-1 interact through epigenetic modifications to regulate coronary artery disease gene expression
Source: Genome Med. 2019 May 2;11:23. doi: 10.1186/s13073-019-0635-9 (PMC6480881; doi:10.1186/s13073-019-0635-9)
Supplement: Supplementary file 1 — Supplemental figures and related figure legends. (PDF 2496 kb) [file 13073_2019_635_MOESM1_ESM.pdf]

Zhao et. al., “”TCF21 and AP-1 interact through epigenetic modifications to regulate coronary artery disease gene expression

Supplemental Figure Legends

Additional file 1: Fig. S1. Knockdown of *JUN* and *TCF21* in HCASMC, and *SMAD3* and *CDKN2BAS* loci regions of study. A) Confirmation of *JUN* or *TCF21* knockdown and B) *TCF21* over-expression evaluated by qPCR of expression levels with *ACTB* normalization. (mean ± SD; *n*=3) C) Relative GAPDH normalized densities for blots shown in Fig. 1D. D) Regions of the *SMAD3* locus, and E) the *CDKN2BAS* locus employed in ChIP-qPCR studies are shown. Presumed AP-1 and TCF21 binding sites are also indicated.

Additional file 2: Fig. S2. AP-1 recruits p300 to mediate H3K27ac histone modification and TCF21 binding. A) Over-expressed TCF21 protein levels evaluated by western blotting of experiment shown in Fig. 2B, and B) the relative GAPDH normalized band densities for TCF21 quantification. C) HCASMC were transfected with p300 siRNA. D) HCASMC were transfected with p300 siRNA alone (p300-KD), transduced with pWPI lentivirus encoding human *JUN* (JUN-OE), or underwent JUN-OE and p300 KD and underwent ChIP-qPCR for H3K27ac histone modification, or E) TCF21 ChIP-PCR. (mean ± SD; *n*=3)

Additional file 3: Fig. S3. TCF21 expression does not affect JUN binding. A) JUN binding with *TCF21* knockdown (TCF21-KD), *TCF21* over-expression (TCF21-OE) or control (Ctrl) transfections in HCASMC. B) TCF21 binding at *SMAD3-1, SMAD3-2* and *CDKN2BAS* locus regions was evaluated by ChIP-qPCR with *TCF21* knockdown (TCF21-KD). (mean ± SD; *n*=3)

Additional file 4: Fig. S4. Genome-wide co-localization of JUN and TCF21 binding. Co-localization of TCF21 and JUN position weight matrix scores on A) JUN peaks or B) TCF21 peaks are shown as a density plot. PWMs were counted in a window of 200 bp and were shown as percentage. C) Heatmap distribution of HNF1A ChIPseq centered on JUN peaks within a 4-kb window. D) Histogram showing the distance distribution from TCF21 to JUN peaks (Green) and JUN to TCF21 peaks (Red) in HCAMSC, and TCF21 to HNF1A peaks (Grey) in HepG2, in the common open chromatin regions of the two cell types. The median distances are indicated with dashed lines (9709 bp, 11372 bp, and 7576 bp median values, respectively) E) Histogram showing the distance distribution of TCF21 to JUN peaks (Green), JUN to TCF21 peaks (Red) in HCAMSC, and TCF21 to HNF1A peaks (Grey) in HepG2 in the HCASMC unique open chromatin region compared to ENCODE data. The median distances are indicated with dashed lines, 12781, 40251, 78559 bp, out of graph range) F) Venn diagram showing the number of overlaps between TCF21 regulated (increased in knockdown, q<1e-10) and JUN regulated H3K27 peaks (decreased in knockdown, q<1e-5) in HCASMC. G) Venn diagram showing the number of overlaps between JUN-TCF21 regulated H3K27ac peaks (from A) and TCF21 or JUN ChIPseq peaks (q<0.01) in HCASMC. H) Biological processes and I) KEGG pathways from DAVID Gene Ontology analysis of JUN-TCF21 regulated H3K27ac genes. Genes were assigned by GREAT with “single nearest” mode.

Additional file 5: Fig. S5. JUN-TCF21 regulated open chromatin regions at CAD loci. A) Venn diagram showing the number of overlaps between JUN regulated ATACseq open chromatin and H3K27 peaks (decreased in knockdown, q<0.01) in HCASMC. B) Venn diagram showing the number of overlaps between TCF21 regulated ATACseq open chromatin and H3K27 peaks (increased in knockdown, q<0.01) in HCASMC. C) Homer known motif analysis of JUN plus TCF21 regulated open chromatin regions identified the AP-1 binding sequence as the top motif, and also found enrichment of the binding sequence for TCF21 and its binding partner TCF12. D) Pattern of ATACseq mapping of open chromatin at HCASMC lineage maker loci encoding *ACTA2* and *MYH11*, in the conditions of *JUN* knockdown (JUN-KD1, JUN-KD2),

TCF21 knockdown (TCF21-KD) or *TCF21* over-expression (TCF21-OE). ENCODE layered H3K27ac data are shown as well. E) KEGG pathways from DAVID Gene Ontology analysis of genes located in JUN and TCF21 regulated open chromatin region. Genes were assigned by GREAT with “single nearest” mode. E) Heatmap showing the expression levels of differentially expressed genes located in JUN and TCF21 regulated open chromatin regions with *TCF21* knockdown RNAseq, the expression levels were normalized with TPM.

Additional file 6: Fig. S6. JUN and TCF21 target CAD associated variants. A) Venn diagram showing the number of overlapped variants from HCAMSC eQTL data (q<0.05), GTEx Coronary Artery (CA) eQTL data (p<0.05) or CARDIoGRAMplusC4D SNPs (p<0.05) in JUN plus TCF21 regulated open chromatin regions. B) Biological processes, C) KEGG pathways, and D) GAD disease enrichment of DAVID Gene Ontology analysis of HCAMSC eQTL target genes located in JUN plus TCF21 regulated open chromatin regions. E) Heatmap showing the expression levels of HCAMSC eQTL related differentially expressed genes with *TCF21* knockdown RNAseq. Expression levels were normalized with TPM. F) JUN plus TCF21 regulated open chromatin regions overlapped with GWAS catalog SNPs. G) JUN and TCF21 peaks overlapped with GWAS catalog SNPs. H) Total TCF21 peaks and, I) total JUN peaks overlapped with GWAS catalog SNPs.

Suppl. Fig. 1

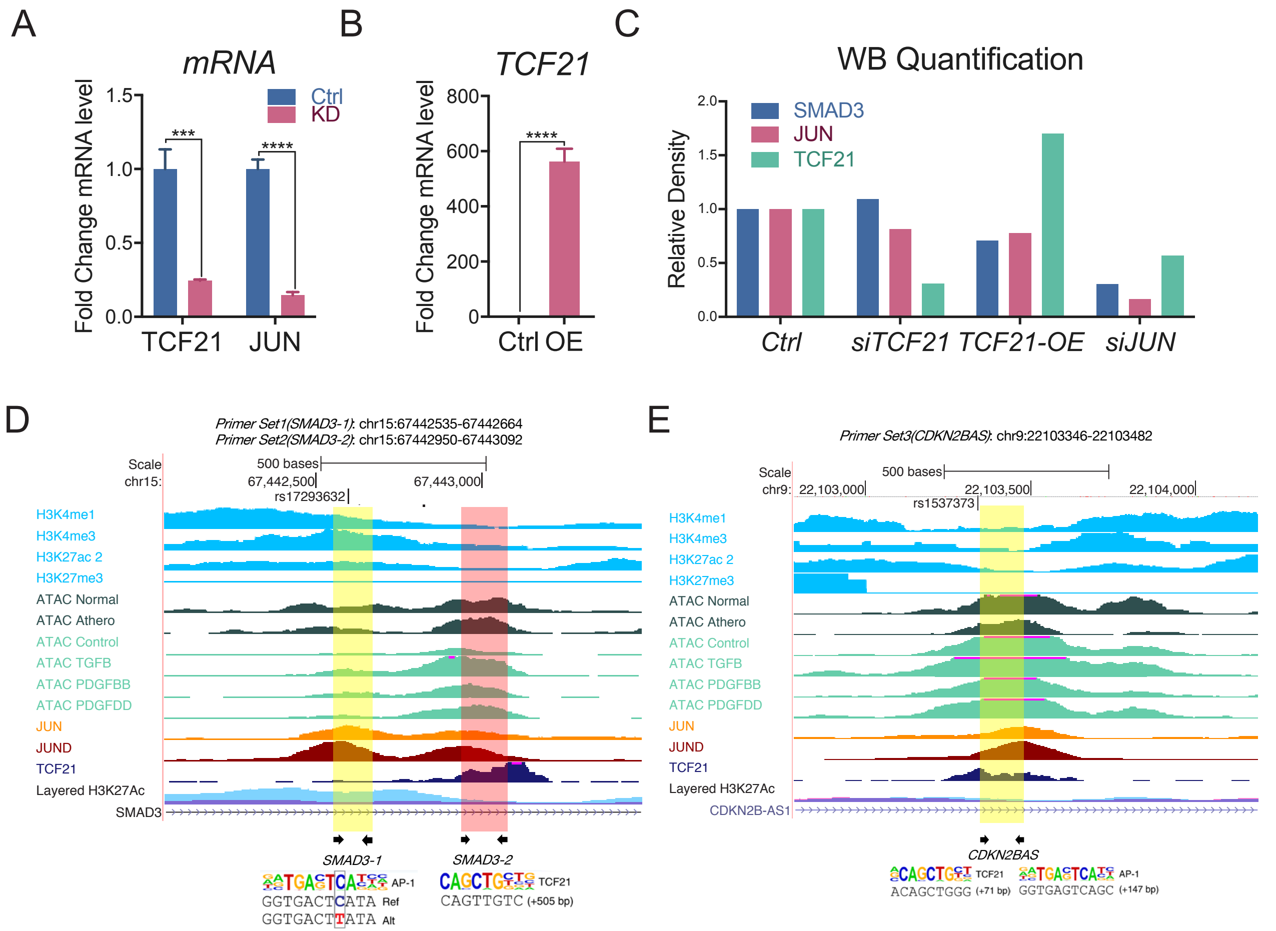

A
mRNA
Fold Change mRNA level
Ctrl
KD
TCF21
JUN
B
TCF21
Ctrl OE
C
WB Quantification
Relative Density
SMAD3
JUN
TCF21
Ctrl
siTCF21
TCF21-OE
siJUN
D
Primer Set1(SMAD3-1): chr15:67442535-67442664
Primer Set2(SMAD3-2): chr15:67442950-67443092
Scale
500 bases
chr15:
67,442,500
67,443,000
rs17293632
H3K4me1
H3K4me3
H3K27ac 2
H3K27me3
ATAC Normal
ATAC Athero
ATAC Control
ATAC TGFB
ATAC PDGFBB
ATAC PDGFDD
JUN
JUND
TCF21
Layered H3K27Ac
SMAD3
SMAD3-1
SMAD3-2
AP-1
GGTGACTCATA Ref
GGTGACTTATA Alt
TCF21
CAGTTGTC (+505 bp)
E
Primer Set3(CDKN2BAS): chr9:22103346-22103482
Scale
500 bases
chr9:
22,103,000
22,103,500
22,104,000
rs1537373
CDKN2B-AS1
CDKN2BAS
TCF21
ACAGCTGGG (+71 bp)
AP-1
GGTGAGTCAGC (+147 bp)

Suppl. Fig. 2

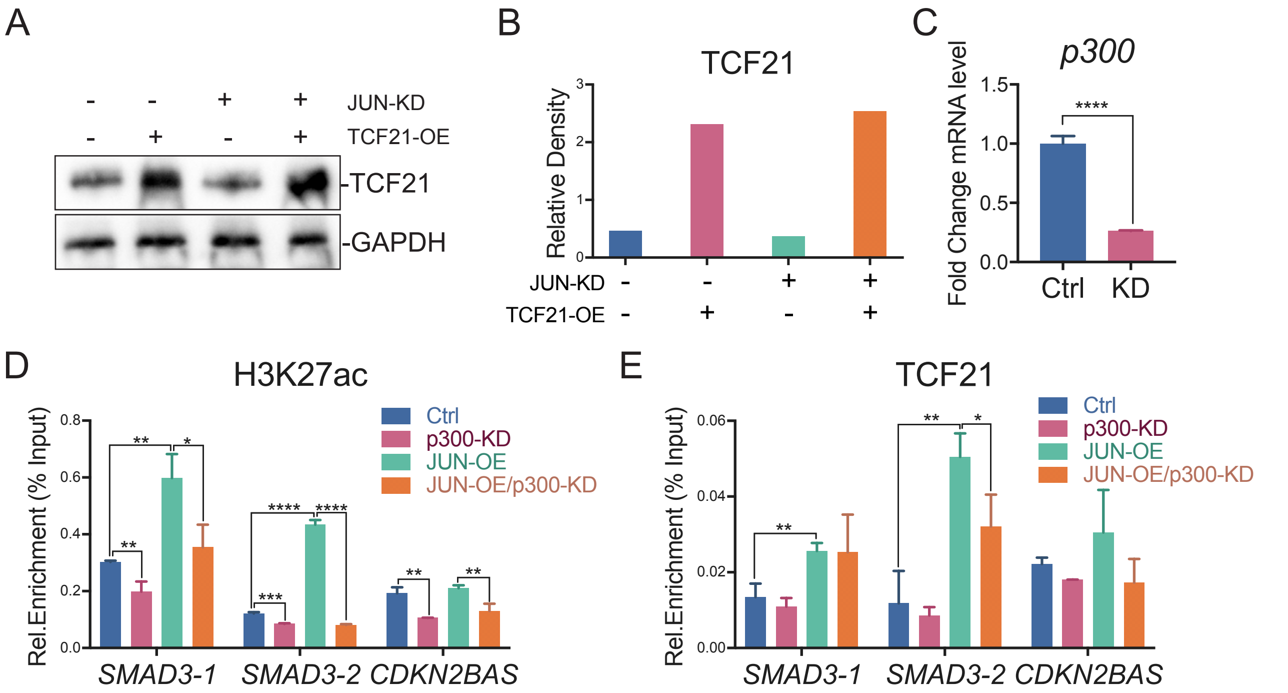

A
- - + + JUN-KD
- + - + TCF21-OE
-TCF21
-GAPDH
B
TCF21
Relative Density
3
2
1
0
JUN-KD - - + +
TCF21-OE - + - +
C
p300
Fold Change mRNA level
1.5
1.0
0.5
0.0
****
Ctrl
KD
D
H3K27ac
Rel.Enrichment (% Input)
0.8
0.6
0.4
0.2
0.0
Ctrl
p300-KD
JUN-OE
JUN-OE/p300-KD
SMAD3-1
SMAD3-2
CDKN2BAS
E
TCF21
Rel.Enrichment (% Input)
0.06
0.04
0.02
0.00
Ctrl
p300-KD
JUN-OE
JUN-OE/p300-KD
SMAD3-1
SMAD3-2
CDKN2BAS

Suppl. Fig. 3

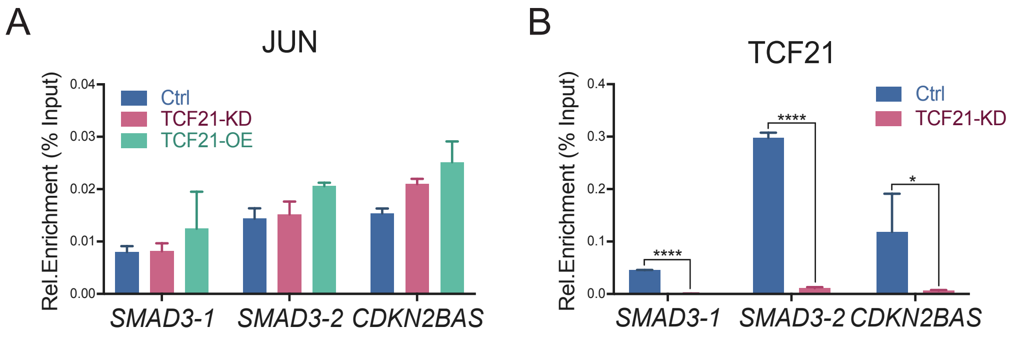
A
JUN
Rel.Enrichment (% Input)
0.04
0.03
0.02
0.01
0.00
Ctrl
TCF21-KD
TCF21-OE
SMAD3-1
SMAD3-2
CDKN2BAS
B
TCF21
Rel.Enrichment (% Input)
0.4
0.3
0.2
0.1
0.0
Ctrl
TCF21-KD
****
****
*
SMAD3-1
SMAD3-2
CDKN2BAS

Suppl. Fig. 4

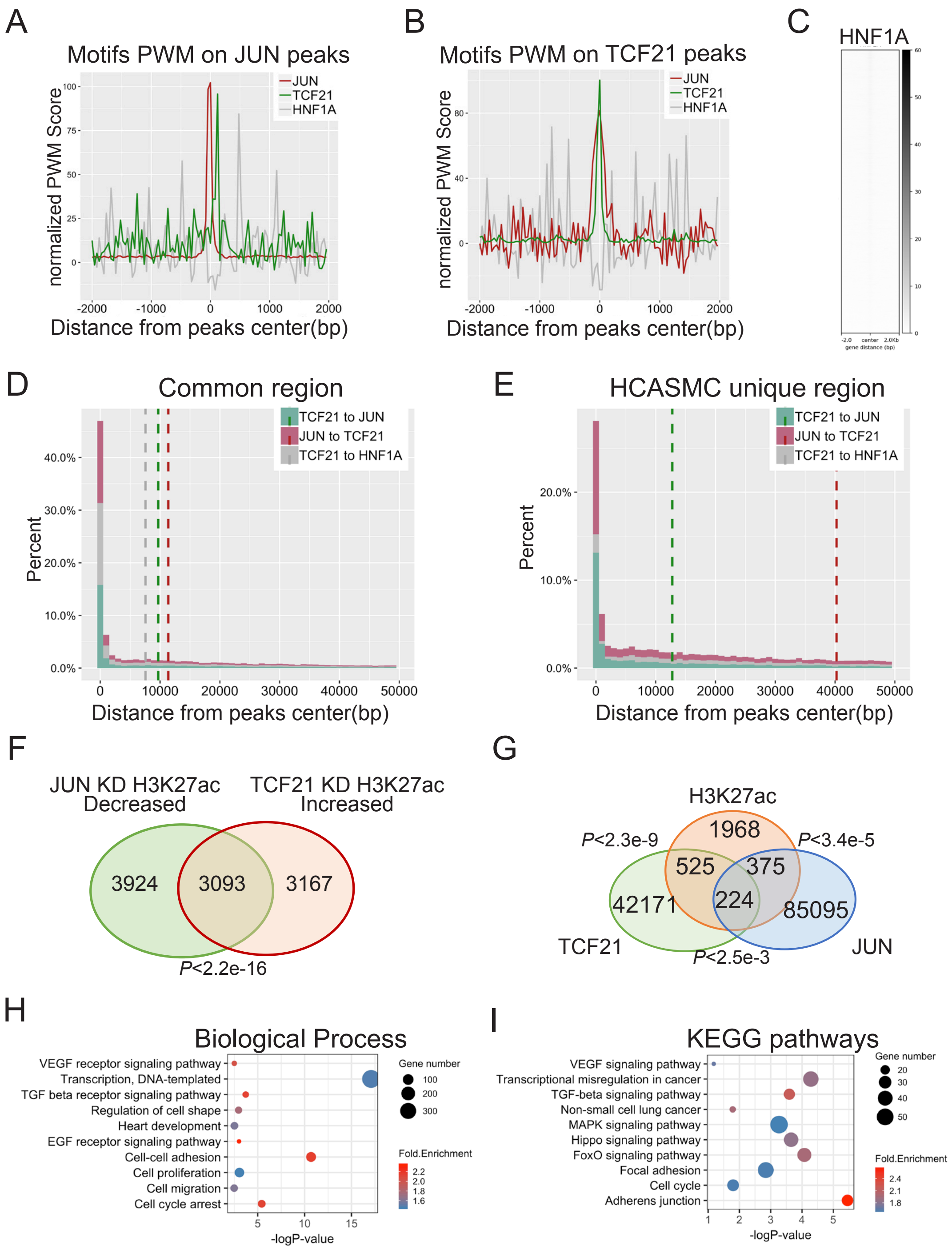
A
Motifs PWM on JUN peaks
JUN
TCF21
HNF1A
normalized PWM Score
Distance from peaks center(bp)
B
Motifs PWM on TCF21 peaks
JUN
TCF21
HNF1A
normalized PWM Score
Distance from peaks center(bp)
C
HNF1A
D
Common region
TCF21 to JUN
JUN to TCF21
TCF21 to HNF1A
Percent
Distance from peaks center(bp)
E
HCASMC unique region
TCF21 to JUN
JUN to TCF21
TCF21 to HNF1A
Percent
Distance from peaks center(bp)
F
JUN KD H3K27ac Decreased
TCF21 KD H3K27ac Increased
3924
3093
3167
P<2.2e-16
G
H3K27ac
1968
P<2.3e-9
P<3.4e-5
525
375
224
42171
85095
TCF21
P<2.5e-3
JUN
H
Biological Process
VEGF receptor signaling pathway
Transcription, DNA-templated
TGF beta receptor signaling pathway
Regulation of cell shape
Heart development
EGF receptor signaling pathway
Cell-cell adhesion
Cell proliferation
Cell migration
Cell cycle arrest
-logP-value
Gene number
Fold.Enrichment
I
KEGG pathways
VEGF signaling pathway
Transcriptional misregulation in cancer
TGF-beta signaling pathway
Non-small cell lung cancer
MAPK signaling pathway
Hippo signaling pathway
FoxO signaling pathway
Focal adhesion
Cell cycle
Adherens junction
-logP-value
Gene number
Fold.Enrichment

Suppl. Fig. 5

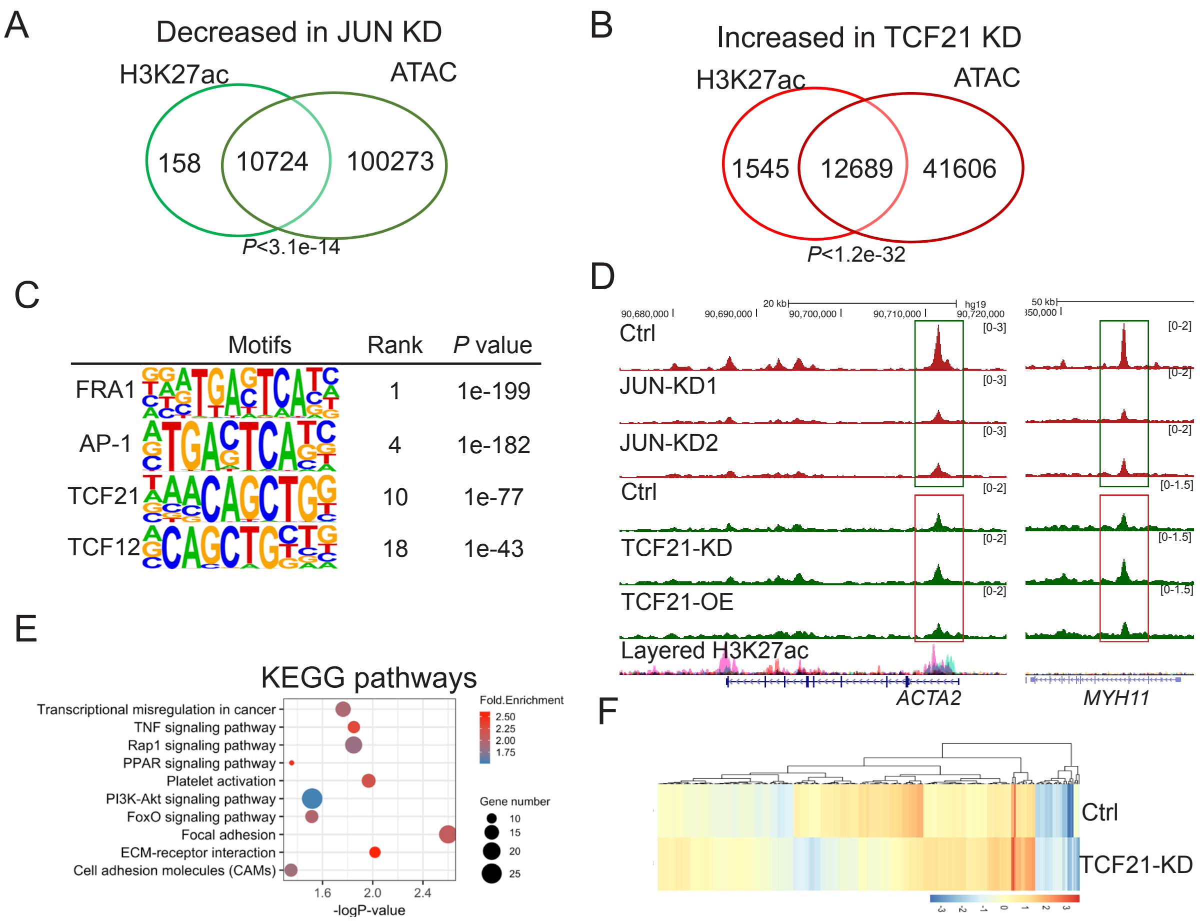
A
Decreased in JUN KD
H3K27ac
ATAC
158
10724
100273
P<3.1e-14
B
Increased in TCF21 KD
H3K27ac
ATAC
1545
12689
41606
P<1.2e-32
C
Motifs
Rank
P value
FRA1
1
1e-199
AP-1
4
1e-182
TCF21
10
1e-77
TCF12
18
1e-43
D
Ctrl
JUN-KD1
JUN-KD2
Ctrl
TCF21-KD
TCF21-OE
Layered H3K27ac
ACTA2
MYH11
E
KEGG pathways
Transcriptional misregulation in cancer
TNF signaling pathway
Rap1 signaling pathway
PPAR signaling pathway
Platelet activation
PI3K-Akt signaling pathway
FoxO signaling pathway
Focal adhesion
ECM-receptor interaction
Cell adhesion molecules (CAMs)
-logP-value
Fold.Enrichment
Gene number
F
Ctrl
TCF21-KD

Suppl. Fig. 6

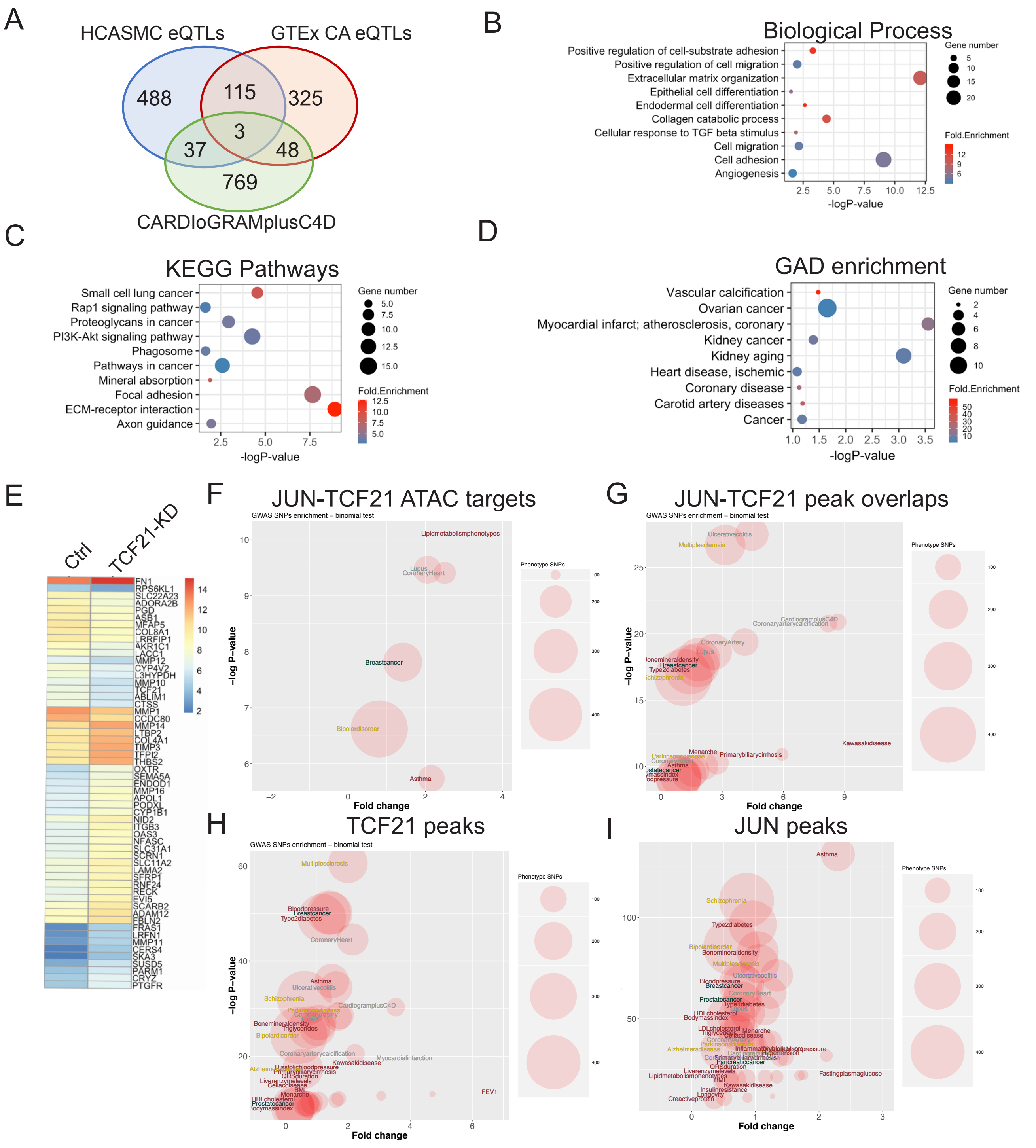
A
HCASMC eQTLs
GTEx CA eQTLs
CARDIoGRAMplusC4D
488
115
325
3
37
48
769
B
Biological Process
Positive regulation of cell-substrate adhesion
Positive regulation of cell migration
Extracellular matrix organization
Epithelial cell differentiation
Endodermal cell differentiation
Collagen catabolic process
Cellular response to TGF beta stimulus
Cell migration
Cell adhesion
Angiogenesis
-logP-value
Gene number
Fold.Enrichment
C
KEGG Pathways
Small cell lung cancer
Rap1 signaling pathway
Proteoglycans in cancer
PI3K-Akt signaling pathway
Phagosome
Pathways in cancer
Mineral absorption
Focal adhesion
ECM-receptor interaction
Axon guidance
-logP-value
Gene number
Fold.Enrichment
D
GAD enrichment
Vascular calcification
Ovarian cancer
Myocardial infarct; atherosclerosis, coronary
Kidney cancer
Kidney aging
Heart disease, ischemic
Coronary disease
Carotid artery diseases
Cancer
-logP-value
Gene number
Fold.Enrichment
E
Ctrl
TCF21-KD
FN1
RPS6KL1
SLC22A23
ADORA2B
PGD
ASB1
MFAP5
COL8A1
LRRFIP1
AKR1C1
LACC1
MMP12
CYP4V2
L3HYPDH
MMP10
TCF21
ABLIM1
CTSS
MMP1
CCDC80
MMP14
LTBP2
COL4A1
TIMP3
TFPI2
THBS2
OXTR
SEMA5A
ENDOD1
MMP16
APOL1
PODXL
CYP1B1
NID2
ITGB3
OAS3
NFASC
SLC31A1
SCRN1
SLC11A2
LAMA2
SFRP1
RNF24
RECK
EVI5
SCARB2
ADAM12
FBLN2
FRAS1
LRFN1
MMP11
CERS4
SKA3
SUSD5
PARM1
CRYZ
PTGFR
F
JUN-TCF21 ATAC targets
GWAS SNPs enrichment – binomial test
Phenotype SNPs
−log P−value
Fold change
G
JUN-TCF21 peak overlaps
GWAS SNPs enrichment – binomial test
Phenotype SNPs
−log P−value
Fold change
H
TCF21 peaks
GWAS SNPs enrichment – binomial test
Phenotype SNPs
−log P−value
Fold change
I
JUN peaks
Phenotype SNPs
−log P−value
Fold change
